# Immune checkpoint blockers plus chemotherapy as the first-line treatment for advanced or metastatic squamous non-small-cell lung carcinoma: a network meta-analysis and economic evaluation

**DOI:** 10.3389/fphar.2025.1669965

**Published:** 2025-10-21

**Authors:** Yitian Lang, Jie Yang, Weican Cao

**Affiliations:** ^1^ Department of Pharmacy, Shanghai Second People’s Hospital, Shanghai, China; ^2^ Department of Pharmacy, Changning Maternity and Infant Health Hospital, East China Normal University, Shanghai, China

**Keywords:** immune checkpoint inhibitors plus chemotherapy, squamous non-small-cell lung cancer, network meta-analysis, economic evaluation, Chinese perspective

## Abstract

**Objective:**

Immune checkpoint inhibitors (ICIs) combined with chemotherapy have shown significant survival benefits in advanced squamous non-small-cell lung cancer (NSCLC), as confirmed by clinical guidelines. However, the high cost of ICIs imposes a substantial economic burden on patients. An economic evaluation of various ICIs plus chemotherapy regimens is urgently needed. This study assessed the cost-effectiveness of several regimens for advanced squamous NSCLC from the perspective of the Chinese healthcare system.

**Methods:**

A network meta-analysis (NMA) was conducted to compare the efficacy of different ICIs plus chemotherapy regimens. The key outcomes, including hazard ratios for overall survival (OS) and progression-free survival (PFS), were extracted from clinical trials. A cost–utility analysis was performed.

**Results:**

Data from six clinical trials involving 2,548 patients were analyzed. The camrelizumab plus chemotherapy and penpulimab plus chemotherapy regimens showed the greatest OS benefit, while camrelizumab plus chemotherapy provided the best PFS benefit. The tislelizumab plus chemotherapy regimen incurred the lowest treatment cost ($42,882.3), with an incremental cost–utility ratio (ICUR) of $ 4,062.0 per quality-adjusted life-year (QALY). The camrelizumab plus chemotherapy regimen offered the highest survival benefit (2.344 QALYs), with an ICUR of $ 6,078.4/QALY. In addition, the ICUR of the penpulimab plus chemotherapy regimen is $25,712.3/QALY. The ICURs of three other ICI plus chemotherapy regimens were higher than the willingness-to-pay threshold.

**Conclusion:**

Among the six ICI plus chemotherapy regimens evaluated, tislelizumab plus chemotherapy demonstrated the lowest ICUR, followed by the camrelizumab plus chemotherapy regimen. However, with a threshold of $13,445/QALY or $40,334/QALY, camrelizumab plus chemotherapy provided greater QALY benefits than tislelizumab plus chemotherapy. Thus, camrelizumab plus chemotherapy is recommended as the preferred first-line treatment for advanced squamous non-small-cell lung cancer in this context.

## 1 Introduction

Globally, lung cancer is the leading cause of cancer mortality, with an estimated incidence of more than 2.48 million cases and approximately 1.81 million deaths in 2022 (Bray et al., 2024). An estimated 714,699 deaths occurred in China, accounting for approximately 40% of global deaths (International Agency for Research on Cancer, 2022).

Non-small-cell lung cancer (NSCLC) accounts for approximately 85% of lung cancer, which includes adenocarcinoma, squamous cell carcinoma, and large-cell carcinoma histological subtypes (Gridelli et al., 2015). The squamous subtype accounts for approximately 30% (Ju et al., 2020; Zhang et al., 2021). Due to the lack of early clinical symptoms and effective screenings, lung cancer is usually diagnosed at an advanced or metastatic stage, resulting in a poor long-term prognosis (Ding et al., 2020). Thus, lung cancer has emerged as a major threat to human health. However, new treatment modalities for cancer continue to emerge, providing additional therapeutic options. To date, the potential of immune checkpoint inhibitors (ICIs) as anti-cancer therapeutics for NSCLC has been continually evaluated. These agents are monoclonal antibodies that target cytotoxic T-lymphocyte-associated antigen-4 (CTLA-4), programmed cell death protein 1 (PD-1), and programmed death ligand 1(PD-L1). A key mechanism by which ICIs function is by sensitizing patient’s own immune system to inhibit cancer cells and prevent immune escape (Yang, 2015; Akkın et al., 2021). Thus, several major breakthroughs have recently been achieved in PD-1/PD-L1 axis immunotherapies. Numerous clinical trials, including the KEYNOTE-407, RATIONALE-307, CameL-Sq, ASTRUM-004, AK105-302, and GEMSTONE-302 trials, have exhibited a significant survival benefit of ICIs in combination with chemotherapy, in terms of overall survival (OS) and progression-free survival (PFS), compared with chemotherapy alone (Ren et al., 2022; Zhou et al., 2022; 2024; Novello et al., 2023; Wang et al., 2024; Zhong et al., 2024). The continuous emergence of novel ICIs provides patients with more treatment options and has gradually formed a new treatment paradigm for advanced squamous NSCLC. However, novel treatment regimens are usually expensive, which imposes heavy economic burdens on patients in many developing countries, such as China.

Cost-effectiveness analysis plays an important role in determining whether new interventions are clinically beneficial at a reasonable cost, with significant implications for public health policies. Clarifying the most cost-effective regimen and the first option among ICIs plus chemotherapy strategies is also meaningful and helpful for clinical oncologists and healthcare decision makers in the setting of finite resources. Due to the absence of head-to-head clinical trials comparing different ICIs plus chemotherapy regimens, we used a network meta-analysis (NMA) approach to establish links between the efficacy, safety, and costs of these ICIs plus chemotherapy regimens. The aim of this study was to assess the cost-effectiveness of several available ICIs plus chemotherapy regimens for advanced squamous NSCLC from the perspective of the Chinese health system.

## 2 Materials and methods

### 2.1 Part I: network meta-analysis

#### 2.1.1 Databases and retrieval strategy

The NMA was performed in accordance with the Preferred Reporting Items for Systematic Reviews and Meta-Analyses (PRISMA) guidelines (Supplementary Table 1) (Hutton et al., 2015). Before the formal literature retrieval, we reviewed the management strategies for NSCLC recommended by the Chinese Society of Clinical Oncology (CSCO) guidelines to identify the ICIs plus chemotherapy regimens. Due to the lack of information regarding the price, we did not analyze ICIs that are not approved in China. Hence, the following agents were determined in the formal retrieval: pembrolizumab (PEM), tislelizumab (TIS), camrelizumab (CAM), serplulimab (SER), penpulimab (PEN), and sugemalimab (SUG). We then retrieved relevant literature studies from PubMed and the Cochrane Library. The retrieval timeframe was limited to 1 January 2000 through 31 December 2024. In addition, the language of publication was restricted to English. Multiple reports of the same clinical trial and single-arm trials were excluded from the network meta-analysis. The detailed retrieval strategy is provided in Supplementary Content 1. We de-duplicated identified literature studies using Zotero (version 7.0.15, https://www.zotero.org/).

#### 2.1.2 Eligibility criteria

This network meta-analysis included studies that met the following PICOS (population, intervention, comparison, outcomes, and study) criteria: clinical trials reporting survival data (including HRs) for ICIs plus chemotherapy versus chemotherapy alone in patients with squamous NSCLC. These ICIs include pembrolizumab, tislelizumab, camrelizumab, sintilimab, serplulimab, penpulimab, and sugemalimab. The exclusion criteria were as follows: (1) patients: younger than 18 years of age or diagnosed with non-squamous NSCLC; (2) intervention: not indicated for first-line therapy, ICI monotherapy regimen, or not approved in China; (3) comparator: non-pharmaceutical treatments or placebo; (4) outcomes: non-survival data or only the PFS or OS data; and (5) study design: non-clinical trial or non-phase-III trial. Additional details regarding the inclusion and exclusion criteria for the network meta-analysis are summarized in Supplementary Table 2.

#### 2.1.3 Review for inclusion

Two authors (YTL and JY) carried out the study selection in a stepwise approach. The titles and abstracts of all studies were independently screened for potential eligibility. Any disagreements were discussed and resolved through consensus. Finally, the eligible studies underwent full-text screening to determine the final set of included studies. The flowchart of study selection is provided in Supplementary Figure 1.

#### 2.1.4 Data extraction

At this stage, two authors (YTL and JY) independently screened the trials and collected data. Data were extracted in an Excel spreadsheet, which included the study name, first author, publication year, sample size, patients, interventions, comparators, median follow-up, and the hazard ratios (HRs) of PFS and OS.

#### 2.1.5 Synthesis of data and statistical analysis

The network meta-analysis was conducted using a Bayesian framework. A fixed-effects model was applied for pooling the HRs of OS and PFS between ICIs and chemotherapy regimens. The values of HR were generated using the R package of “gemtc.” Due to the absence of closed loops in this network, consistency testing was not applicable. The risk of bias for these included studies was assessed in RevMan software (version 5.4, https://training.cochrane.org/online-learning/core-software/revman). The traffic-light plot is shown in Supplementary Figure 2.

### 2.2 Part II: cost-effectiveness analysis

#### 2.2.1 Model structure

A decision analytic model was developed to evaluate the cost-effectiveness of six ICIs plus chemotherapy regimens for the treatment of squamous NSCLC from the perspective of the Chinese healthcare system. A partitioned survival approach (PartSA) was applied to simulate the disease development of patients with squamous NSCLC. The simulated population for each regimen was matched to the patients in the KEYNOTE-407, RATIONALE-307, CameL-Sq, ASTRUM-004, AK105-302, and GEMSTONE-302 trials.

In this PartSA, there are three mutually exclusive health states, namely, PFS, progressive disease (PD), and death. Patients were assumed to enter the model in the PFS state, which could transition to the PD state or death state, based on clinical survival data.

In this analysis, the patients with NSCLC received the following therapy regimens: (1) pembrolizumab plus chemotherapy (PEMC), (2) tislelizumab plus chemotherapy (TISC), (3) camrelizumab plus chemotherapy (CAMC), (4) serplulimab plus chemotherapy (SERC), (5) penpulimab plus chemotherapy (PENC), and (6) sugemalimab plus chemotherapy (SUGC). These regimens and the doses strategies were maintained as in the KEYNOTE-407, RATIONALE-307, CameL-Sq, ASTRUM-004, AK105-302, and GEMSTONE-302 trials, respectively. The chemotherapy regimen is primarily composed of carboplatin and taxane. Carboplatin was administered at a dose calculated to produce an area under the concentration–time curve of 5 or 6 mg/mL per minute on day 1 of each 3-week cycle. Paclitaxel was administered at a dose of 175 mg/m^2^ body surface area (BSA) intravenously on day 1 of each 3-week cycle. Nab-paclitaxel was administered at a dose of 100 mg/m^2^ on days 1, 8, and 15 of each 3-week cycle.

**FIGURE 1:**
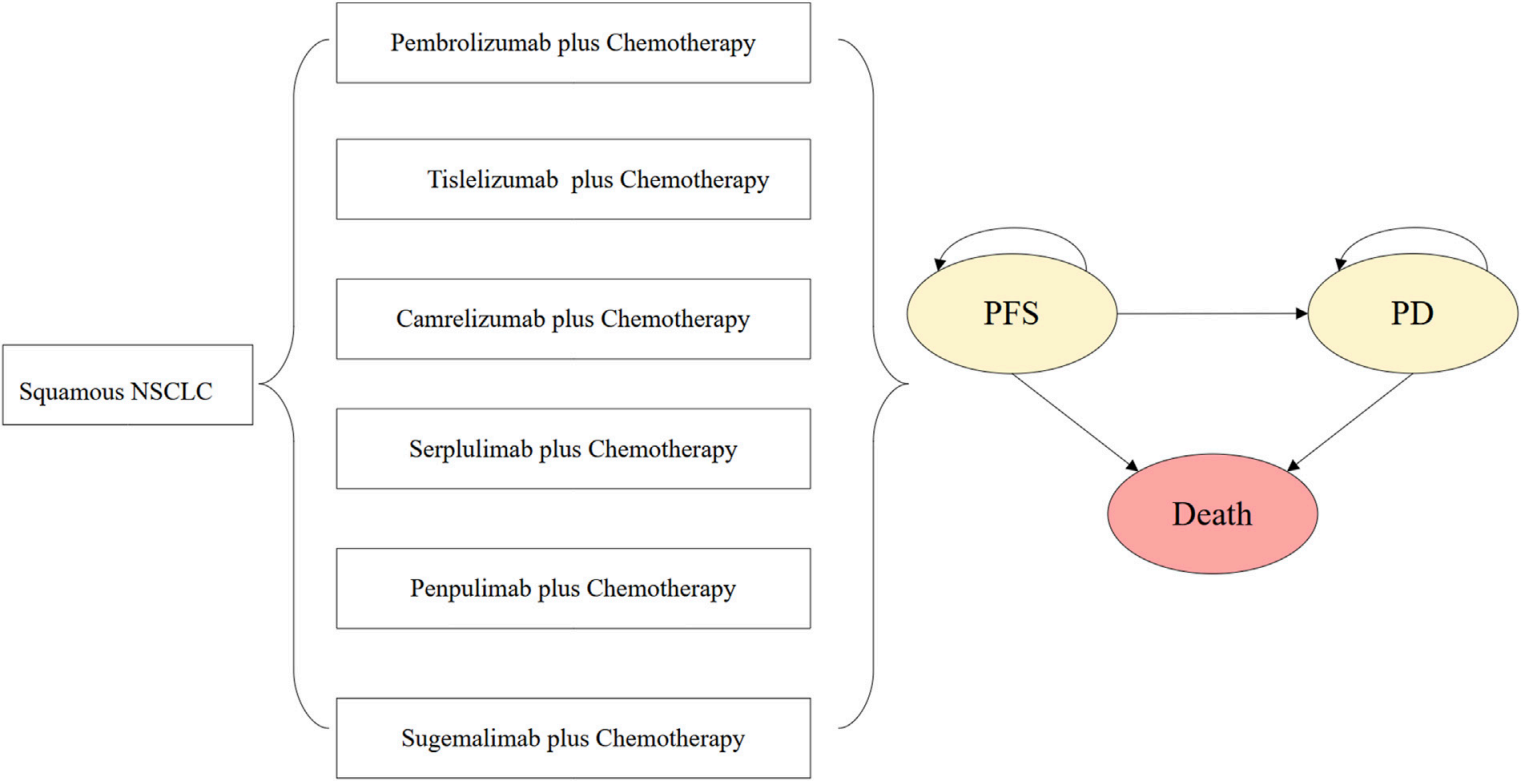
Decision tree and model structure overview. Abbreviations: *NSCLC*, non-small-cell lung cancer; *PFS*, progression-free survival; *PD*, progressive disease.

The dosing details of ICIs were described as follows. Pembrolizumab was administered at a dose of 200 mg on day 1 of each 3-week cycle. Tislelizumab was administered at a dose of 200 mg on day 1 of each 3-week cycle. Camrelizumab was administered at a dose of 200 mg on day 1 of each 3-week cycle. Serplulimab was administered at a dose of 4.5 mg/kg on day 1 of each 3-week cycle. Penpulimab was administered at a dose of 200 mg on day 1 of each 3-week cycle. Sugemalimab was administered at a dose of 1,200 mg on day 1 of each 3-week cycle. The initial treatment was continued until disease progression, unacceptable toxic effects, or death occurred. Once the disease progressed, the patients were assumed to receive the subsequent line therapy and best supportive care. For the purpose of model simplification, docetaxel was selected as the second-line treatment regimen for all ICI plus chemotherapy groups in this analysis. Docetaxel was administered at a dose of 75 mg/m^2^ on day 1 of each 3-week cycle. Based on the administration cycle, the 3-week model cycle length was set to facilitate cost estimates. The 10-year time horizon is determined in the analysis. The decision tree and model structure are shown in Figure 1.

#### 2.2.2 Clinical data

The KEYNOTE-407, RATIONALE-407, CameL-Sq, ASTRUM-004, AK105-302, and GEMSTONE-302 trials reported the PFS, OS data, and safety data. However, the observed follow-up time was insufficient to cover the entire model time horizon. Therefore, appropriate extrapolation beyond the follow-up time is needed. The PFS and OS curves from each of the aforementioned trials were digitized to extract time-to-survival data. To obtain the time-to-event data, this study employed the algorithm developed by Guyot et al. (2012) to generate pseudo-individual participant data (IPD) . The generated time-to-event data were fitted to a range of parametric distributions, including Weibull, Gompertz, exponential, log-normal, and log-logistic distributions. The Akaike information criterion (AIC) value of each distribution for all arms was calculated, and the best-fitted distribution was determined according to the AIC values and visual inspection. The best-fitted distributions and curve parameters are shown in Table 1.

**TABLE 1:** Key model inputs: base-case values, ranges, and distribution.

Parameter	Distribution	Value (range), USD	References
Survival input			
Projected model of chemotherapy for PFS	Log-logistic	Shape = 2.0776 Scale = 7.1862	Ren et al. (2022), Zhou et al. (2022), 2024; Novello et al. (2023), Wang et al. (2024), Zhong et al. (2024)
Projected model of chemotherapy for OS	Log-logistic	Shape = 1.5621 Scale = 23.0596	Ren et al. (2022), Zhou et al. (2022), 2024; Novello et al. (2023), Wang et al. (2024), Zhong et al. (2024)
HRs of ICI plus chemotherapy regimens against chemotherapy alone	Log-normal	See the results of NMA	NMA
Treatment cost			
Pembrolizumab (per 100 mg)	Gamma	2,516 (1,887–3,145)	Sunshine Medical Procurement All-In-One (2024)
			
Tislelizumab (per 100 mg)	Gamma	176 (132–220)	Sunshine Medical Procurement All-In-One (2024)
Camrelizumab (per 200 mg)	Gamma	362 (271.5–452.5)	Sunshine Medical Procurement All-In-One (2024)
Serplulimab (per 100 mg)	Gamma	785 (588.8–981.3)	Sunshine Medical Procurement All-In-One (2024)
Penpulimab (per 100 mg)	Gamma	501 (375.8–626.3)	Sunshine Medical Procurement All-In-One (2024)
Sugemalimab (per 600 mg)	Gamma	1,738 (1,303.5–2,172.5)	Sunshine Medical Procurement All-In-One (2024)
Carboplatin (per 100 mg)	Gamma	5.8 (4.4–7.3)	Sunshine Medical Procurement All-In-One (2024)
Paclitaxel (per 100 mg)	Gamma	9.5 (7.1–11.9)	Sunshine Medical Procurement All-In-One (2024)
Nab-paclitaxel (per 100 mg)	Gamma	92.7 (69.5–115.9)	Sunshine Medical Procurement All-In-One (2024)
Docetaxel (per 80 mg)	Gamma	8.3 (6.2–10.4)	Sunshine Medical Procurement All-In-One (2024)
Administration (per cycle)	Gamma	61.72 (46.29–77.15)	Liu et al. (2022)
Follow-up (per cycle)	Gamma	59.2 (44.4–74)	Kang et al. (2022)
Best supportive care (per cycle)	Gamma	1,415.02 (1,061.27–1,768.78)	Liu et al. (2022)
Palliative care	Gamma	2,099.15 (1,574.36–2,623.94)	Liu et al. (2022)
Expenditure of AE management			
Anemia	Gamma	468.19 (351.14–585.24)	Chen et al. (2022)
Neutropenia	Gamma	681.91 (511.43–852.39)	Chen et al. (2022)
Diarrhea	Gamma	29 (21.75–36.25)	Kang et al. (2022)
White blood cell count decreased	Gamma	115.01 (86.26–143.76)	Chen et al. (2022)
Platelet count decreased	Gamma	1,505.92 (1,129.44–1,882.4)	Chen et al. (2022)
Pneumonia	Gamma	6491.17 (4,868.38–8,113.96)	Shao et al. (2022)
Pooled incidence of severe AEs in the chemotherapy arm			
Anemia	Beta	11.7% (8.8%–14.6%)	(Ren et al., 2022; Zhou et al., 2022; 2024; Novello et al., 2023; Wang et al., 2024; Zhong et al., 2024)
Neutropenia	Beta	12.7% (9.5%–15.9%)	(Ren et al., 2022; Zhou et al., 2022; 2024; Novello et al., 2023; Wang et al., 2024; Zhong et al., 2024)
Diarrhea	Beta	0.8% (0.6%–1.0%)	(Ren et al., 2022; Zhou et al., 2022; 2024; Novello et al., 2023; Wang et al., 2024; Zhong et al., 2024)
White blood cell count decreased	Beta	10.4% (7.8%–13.0%)	(Ren et al., 2022; Zhou et al., 2022; 2024; Novello et al., 2023; Wang et al., 2024; Zhong et al., 2024)
Platelet count decreased	Beta	3.2% (2.4%–4.0%)	(Ren et al., 2022; Zhou et al., 2022; 2024; Novello et al., 2023; Wang et al., 2024; Zhong et al., 2024)
Pneumonia	Beta	2.0% (1.5%–2.5%)	(Ren et al., 2022; Zhou et al., 2022; 2024; Novello et al., 2023; Wang et al., 2024; Zhong et al., 2024)
Incidence of severe AEs in the pembrolizumab combination arm			
Anemia	Beta	15.8% (11.9%–19.8%)	Novello et al. (2023)
Neutropenia	Beta	23.0% (17.3%–28.8%)	Novello et al. (2023)
Diarrhea	Beta	4.3% (3.2%–5.4%)	Novello et al. (2023)
Pneumonia	Beta	3.2% (2.4%–4.0%)	Novello et al. (2023)
Incidence of severe AEs in the tislelizumab combination arm			
Anemia	Beta	10.0% (7.5%–12.5%)	Wang et al. (2024)
Neutropenia	Beta	33.3% (25.0%–41.6%)	Wang et al. (2024)
White blood cell count decreased	Beta	23.3% (17.5%–29.1%)	Wang et al. (2024)
Platelet count decreased	Beta	5.0% (3.8%–6.3%)	Wang et al. (2024)
Pneumonia	Beta	5.0% (3.8%–6.3%)	Wang et al. (2024)
Incidence of severe AEs in the camrelizumab combination arm			
Anemia	Beta	3.1% (2.3%–3.9%)	Ren et al. (2022)
Neutropenia	Beta	3.1% (2.3%–3.9%)	Ren et al. (2022)
Diarrhea	Beta	2.3% (1.7%–2.9%)	Ren et al. (2022)
White blood cell count decreased	Beta	2.1% (1.6%–2.6%)	Ren et al. (2022)
Platelet count decreased	Beta	2.1% (1.6%–2.6%)	Ren et al. (2022)
Pneumonia	Beta	3.6% (2.7%–4.5%)	Ren et al. (2022)
Incidence of severe AEs in the serplulimab combination arm			
Anemia	Beta	12.0% (9.0%–15.0%)	Zhou et al. (2024)
Neutropenia	Beta	4.7% (3.5%–5.9%)	Zhou et al. (2024)
White blood cell count decreased	Beta	10.1% (7.6%–12.6%)	Zhou et al. (2024)
Platelet count decreased	Beta	6.7% (5.0%–8.4%)	Zhou et al. (2024)
Pneumonia	Beta	1.4% (1.1%–1.8%)	Zhou et al. (2024)
Incidence of severe AEs in the penpulimab combination arm			
Anemia	Beta	1.7% (1.3%–2.1%)	Zhong et al. (2024)
White blood cell count decreased	Beta	20.2% (15.2%–25.3%)	Zhong et al. (2024)
Platelet count decreased	Beta	3.5% (2.6%–4.4%)	Zhong et al. (2024)
Incidence of severe AEs in the sugemalimab combination arm			
Anemia	Beta	13.4% (10.1%–16.8%)	Zhou et al. (2022)
Neutropenia	Beta	3.8% (2.9%–4.8%)	Zhou et al. (2022)
Diarrhea	Beta	0.9% (0.7%–1.1%)	Zhou et al. (2022)
White blood cell count decreased	Beta	14.1% (10.6%–17.6%)	Zhou et al. (2022)
Platelet count decreased	Beta	10.3% (7.7%–12.9%)	Zhou et al. (2022)
Pneumonia	Beta	1.9% (1.4%–2.4%)	Zhou et al. (2022)
Utility estimate			
Progression-free disease	Beta	0.82 (0.615–1)	Lu et al. (2017)
Progressive disease	Beta	0.58 (0.435–0.725)	Lu et al. (2017)
Utility decrement			
Anemia	Beta	0.12 (0.09–0.15)	Wheeler et al. (2022)
Neutropenia	Beta	0.09 (0.0675–0.1125)	Wheeler et al. (2022)
Diarrhea	Beta	0.07 (0.0525–0.0875)	Nafees et al. (2017)
White blood cell count decreased	Beta	0.20 (0.15–0.25)	Shao et al. (2022)
Platelet count decreased	Beta	0.11 (0.0825–0.1375)	Shao et al. (2022)
Pneumonia	Beta	0.05 (0.0375–0.0625)	Shao et al. (2022)
Other parameters			
Weight	Normal	65 (48.8–81.3)	Lu et al. (2017)
Body surface area, m^2^	Normal	1.72 (1.5–1.9)	Lu et al. (2017)

The costs of AEs in this table are presented on a per-event basis, and the costs and disutilities of AEs were calculated only once at the start of the analysis model. All costs sourced from China in this study were converted into US dollars ($1 = RMB 7.1217, average exchange rate for 2024). All costs reported for years prior to 2024 were updated to 2024 USD.

*Abbreviations: HRs*, hazard ratios; *ICI*, immune checkpoint inhibitor; *NMA*, network meta-analysis; *PFS*, progression-free survival; *OS*, overall survival; *AEs*, adverse events, *USD*, US dollars.

Since grade 1 to 2 treatment-related adverse events (AEs) can be effectively managed, only grade 3 and 4 AEs were included in the analysis. The incidence of AEs is summarized in Table 1.

#### 2.2.3 Costs and utilities

The Chinese healthcare system perspective was adopted in this analysis. Therefore, direct medical expenditures were included in the cost estimates, which covered the therapy drugs, administration for intravenous injection, management of severe AEs, follow-up, and palliative care. Costs of drugs were obtained from a local charge database (Sunshine Medical Procurement All-In-One, 2024). An average BSA of 1.72 m^2^ and an average body weight of 65 kg were used for dosing estimates in this analysis (Lu et al., 2017). The overall drug costs were calculated according to the predetermined dosing strategy. The costs of intravenous administration, palliative care, follow-up, and best supportive care were obtained from published studies (Liu et al., 2022). The expenditure of the management of severe AEs (grade 3 and above) was gathered from associated economic studies (Chen et al., 2022; Kang et al., 2022; Shao et al., 2022). All costs presented for years prior to 2024 were adjusted to 2024 US dollars (USD).

Each health state in the PartSA model was assigned a health utility value reflecting both the disease progression stage and the impact of severe AEs as both factors are critical for the quality of life (QoL) for patients with advanced or metastatic squamous NSCLC. We assumed that QoL is linked to two key components: the progressive stage of the disease and severe AEs. The base utility value for the PFS state was estimated to be 0.82, and the PD state was estimated to be 0.58, with these values derived from published studies to reflect the core impact of the disease stage (Lu et al., 2017). To account for the negative QoL effects of severe AEs, these base utility values are further adjusted by utility decrements specific to severe AEs. The decrement values for these AEs were sourced from previous published research, ensuring that we incorporate both disease- and treatment-related impacts into our QALY calculations (Nafees et al., 2017; Wheeler et al., 2022). More detailed values of the inputs are summarized in Table 1.

#### 2.2.4 Analyses

In the base-case analysis, the ICUR was used to assess the incremental cost per QALY. In addition, life-years (LYs) gained were also measured to assess the total survival benefit. All QALYs and costs were discounted at an annual rate of 5%. It indicates that the regimen is “cost-effective” if the ICUR is below the willingness-to-pay (WTP) threshold. In China, the WTP threshold was set at thrice the *per capita* gross domestic product (GDP, calculated to be $ 40,334) in 2024. If multiple regimens fall below this threshold, a further evaluation using a threshold of one times the *per capita* GDP (calculated to be $ 13,445) should be conducted to determine which regimen is more cost-effective.

A series of uncertainty analyses, including one-way deterministic sensitivity analyses (DSAs) and probabilistic sensitivity analyses (PSAs), were performed to test the model’s robustness. DSAs were applied to assess the impact of the uncertainty of a single input on the ICUR. The range of annual discount rates is from 0% to 8%. Other model inputs were assumed to have a variation within the reported 95% confidence intervals (CI) or reasonable ranges (±25% of the base-case value). In the PSAs, Monte Carlo simulations of 1,000 iterations were generated by simultaneously sampling the key parameters based on pre-specified probability distributions. All inputs related to costs were assigned gamma distributions, and the inputs linked to the incidence of AEs and utilities were assigned beta distributions (Briggs et al., 2012). The cost-effectiveness acceptability curve (CEAC) and scatterplot were generated to clearly present the likelihood that the treatment strategy was regarded as ‘cost-effective’ at a range of thresholds. All analyses, including PartSA and the cost-effectiveness model, were programmed and conducted in R software (version 4.4.2, http://www.r-project.org).

## 3 Results

### 3.1 Part I: network meta-analysis

#### 3.1.1 Studies included and the risk of bias

After screening the titles and abstracts, six clinical trials involving 2,548 patients were included in the final analysis. The network plot of evidence from all trials is shown in Supplementary Figure 2. The summary of the trial characteristics is provided in Supplementary Table 3. Patients received one of the following first-line treatment regimens: pembrolizumab plus chemotherapy (n = 278 patients), tislelizumab plus chemotherapy (n = 120 patients), camrelizumab plus chemotherapy (n = 193 patients), serplulimab plus chemotherapy (n = 358 patients), penpulimab plus chemotherapy (n = 173 patients), sugemalimab plus chemotherapy (n = 320 patients), or placebo plus chemotherapy (n = 1,106 patients). The risk of bias assessments for the included trials are presented as a traffic-light plot in Supplementary Figure 3. The indirect comparison HRs for PFS and OS among the ICIs are summarized in Table 2.

**TABLE 2:** Hazard ratios (95% CI) of the network meta-analysis of the progression-free survival and overall survival.

PFS	PEMC	TISC	CAMC	SERC	PENC	SUGC	C
PEMC	1	1.4 (0.96, 2.0)	1.7 (1.2, 2.3)	1.2 (0.87, 1.6)	1.4 (1.0, 2.0)	1.3 (0.98, 1.7)	0.62 (0.52, 0.74)
TISC	0.73 (0.51, 1.0)	1	1.2 (0.82, 1.8)	0.85 (0.58, 1.3)	1.0 (0.69, 1.6)	0.94 (0.64, 1.4)	0.45 (0.33, 0.62)
CAMC	0.60 (0.44, 0.81)	0.82 (0.56, 1.2)	1	0.70 (0.50, 0.98)	0.86 (0.60, 1.2)	0.77 (0.56, 1.1)	0.37 (0.29, 0.47)
SERC	0.85 (0.64, 1.1)	1.2 (0.80, 1.7)	1.4 (1.0, 2.0)	1	1.2 (0.87, 1.8)	1.1 (0.80, 1.5)	0.53 (0.42, 0.67)
PENC	0.69 (0.51, 0.96)	0.96 (0.63, 1.4)	1.2 (0.81, 1.7)	0.81 (0.57, 1.1)	1	0.90 (0.64, 1.3)	0.43 (0.33, 0.56)
SUGC	0.78 (0.59, 1.0)	1.1 (0.73, 1.6)	1.3 (0.94, 1.8)	0.91 (0.66, 1.2)	1.1 (0.80,1.6)	1	0.48 (0.39, 0.60)
C	1.60 (1.40, 1.90)	2.2 (1.6, 3.1)	2.7 (2.1, 3.4)	1.9 (1.5, 2.4)	2.3 (1.8, 3.0)	2.1 (1.7, 2.6)	1
OS	PEMC	TISC	CAMC	SERC	PENC	SUGC	C
PEMC	1	1.0 (0.71, 1.50)	1.3 (0.90, 1.9)	0.97 (0.72, 1.3)	1.3 (0.89, 1.9)	1.1 (0.75, 1.5)	0.71 (0.59, 0.85)
TISC	0.97 (0.67, 1.4)	1	1.3 (0.81, 2.0)	0.95 (0.64, 1.4)	1.3 (0.81,2.0)	1.0 (0.66, 1.6)	0.69 (0.50, 0.95)
CAMC	0.77 (0.54, 1.1)	0.79 (0.51, 1.2)	1	0.75 (0.51, 1.1)	1.0 (0.64,1.6)	0.82 (0.53, 1.3)	0.55 (0.40, 0.75)
SERC	1.0 (0.76, 1.4)	1.1 (0.71, 1.6)	1.3 (0.89, 2.0)	1	1.3 (0.89, 2.0)	1.1 (0.75, 1.6)	0.73 (0.58, 0.92)
PENC	0.77 (0.54, 1.1)	0.80 (0.51, 1.2)	1.0 (0.64, 1.6)	0.75 (0.51, 1.1)	1	0.82 (0.53, 1.3)	0.55 (0.40, 0.75)
SUGC	0.95 (0.67, 1.3)	0.97 (0.63, 1.5)	1.2 (0.79, 1.9)	0.92 (0.63, 1.3)	1.2 (0.79, 1.9)	1	0.67 (0.50, 0.90)
C	1.4 (1.2, 1.7)	1.4 (1.1, 2.0)	1.8 (1.3, 2.5)	1.4 (1.1, 1.7)	1.8 (1.3, 2.5)	1.5 (1.1, 2.0)	1

Abbreviations: *PEMC*, pembrolizumab plus chemotherapy; *TISC*, tislelizumab plus chemotherapy; *CAMC*, camrelizumab plus chemotherapy; *SERC*, serplulimab plus chemotherapy; *PENC*, penpulimab plus chemotherapy; *SUGC*, sugemalimab plus chemotherapy; *C*, chemotherapy; *PFS*, progression-free survival; *OS*, overall survival; *CI*, confidence interval.

### 3.2 Part II: curve fitting

The survival curve represents a progression from healthier to sicker states, ending in death. The survival functions generally should not cross. In this analysis, the distributions that led to crossovers between OS and PFS curves were not included. Combining AIC, BIC, and visual inspection, the log-logistic distribution appears to be the most rational function for extrapolating PFS and OS of the chemotherapy regimen. Reconstructed Kaplan–Meier survival curves based on pooled time-to-event data for the chemotherapy regimen are shown in Supplementary Figure 4. The scale and shape parameters of the projected curve of the chemotherapy arm are provided in Supplementary Table 4. The projected survival curves of each ICI plus chemotherapy regimen were generated based on the hazard ratios from the network meta-analysis and are shown in Supplementary Figure 5. The results for the proportion of patients at each time point are shown in Supplementary Figure 6.

### 3.3 Part III: cost-effectiveness analysis

#### 3.3.1 Base-case analysis

Patients who received the chemotherapy regimen experienced a gain of 1.966 LYs and 1.252 QALYs with an associated cost of $40,583.2. Meanwhile, patients who underwent the pembrolizumab plus chemotherapy regimen achieved a gain of 2.683 LYs and 1.761 QALYs at a cost of $117,423.1. Those on the tislelizumab plus chemotherapy regimen gained 2.748 LYs and 1.818 QALYs with a cost of $42,882.3. The camrelizumab plus chemotherapy regimen resulted in 3.284 LYs and 2.344 QALYs at a cost of $47,220.8. The serplulimab plus chemotherapy regimen yielded 2.620 LYs and 1.766 QALYs with an associated cost of $77,714.8. The penpulimab plus chemotherapy regimen yielded 3.284 LYs and 2.224 QALYs at a cost of $65,575.6. Patients on the sugemalimab plus chemotherapy regimen gained 2.816 LYs and 1.892 QALYs with a cost of $97,293.8.

Compared to the chemotherapy regimen, the pembrolizumab plus chemotherapy regimen incurred an additional cost of $76,839.9, with an increase of 0.717 LYs and 0.509 QALYs, resulting in an ICUR of $150,962.4/QALY. The tislelizumab plus chemotherapy regimen incurred an additional cost of $2,299.1 relative to the chemotherapy regimen, along with an incremental effectiveness of 0.782 LYs and 0.566 QALYs, leading to an ICUR of $4,062/QALY. For the camrelizumab plus chemotherapy regimen versus the chemotherapy regimen, the additional cost was $6,637.6, with an increase of 1.318 LYs and 1.092 QALYs, corresponding to an ICUR of $6,078.4/QALY.

The serplulimab plus chemotherapy regimen, compared to the chemotherapy regimen, incurred an additional cost of $37,131.6 and an incremental effectiveness of 0.654 LYs and 0.514 QALYs, resulting in an ICUR of $72,240.4/QALY. Relative to the chemotherapy regimen, the penpulimab plus chemotherapy regimen incurred an additional cost of $24,992.4 and an increase of 1.318 LYs and 0.972 QALYs, with an ICUR of $25,712.3/QALY. The sugemalimab plus chemotherapy regimen, compared to the chemotherapy regimen, incurred an additional cost of $56,710.6, with an incremental effectiveness of 0.85 LYs and 0.640 QALYs, leading to an ICUR of $88,610.3/QALY. Detailed results of the base-case analysis are summarized in Table 3.

**TABLE 3:** Results of the base-case analysis.

Regimen	Chemotherapy	PEMC	TISC	CAMC	SERC	PENC	SUGC
Total cost	$40,583.2	$117,423.1	$42,882.3	$47,220.8	$77,714.8	$65,575.6	$97,293.8
By item							
Medication	$1,167.7	$70,860.7	$8,174.3	$9,252.5	$39,166.6	$19,411.3	$57,891.3
AE management	$328.9	$437.5	$698.0	$303.1	$290.4	$83.6	$382.1
Subsequent treatment	$39,086.7	$46,124.9	$34,010.1	$37,665.2	$38,257.8	$46,080.6	$39,020.4
By stage							
PFS stage	$3,578.5	$73,386.7	$10,963.6	$11,648.4	$41,546.9	$21,586.6	$60,364.2
PD stage	$37,004.7	$44,036.4	$31,918.7	$35,572.4	$36,167.8	$43,988.9	$36,929.5
Difference (vs. chemotherapy)	-	$76,839.9	$2,299.1	$6,637.6	$37,131.6	$24,992.4	$56,710.6
Total LY	1.966	2.683	2.748	3.284	2.62	3.284	2.816
By stage							
PFS stage	0.592	1.048	1.563	1.963	1.277	1.651	1.445
PD stage	1.374	1.635	1.185	1.321	1.343	1.634	1.371
Difference (vs. chemotherapy)	-	0.717	0.782	1.318	0.654	1.318	0.85
Total QALY	1.252	1.761	1.818	2.344	1.766	2.224	1.892
By stage							
PFS stage	0.455	0.813	1.131	1.577	0.987	1.277	1.097
PD stage	0.797	0.948	0.687	0.766	0.779	0.947	0.795
Difference (vs. chemotherapy)	-	0.509	0.566	1.092	0.514	0.972	0.64
ICUR($/QALY)	-	$150,962.4	$4,062.0	$6,078.4	$72,240.4	$25,712.3	$88,610.3

Abbreviations: PEMC, pembrolizumab plus chemotherapy; TISC, tislelizumab plus chemotherapy; CAMC, camrelizumab plus chemotherapy; SERC, serplulimab plus chemotherapy; PENC, penpulimab plus chemotherapy; SUGC, sugemalimab plus chemotherapy; C, chemotherapy; PFS, progression-free survival; OS, overall survival; QALY, quality-adjusted life-year; *LY*, life-year; *ICUR*, incremental cost–utility ratio.

#### 3.3.2 One-way sensitivity analysis

**FIGURE 2:**
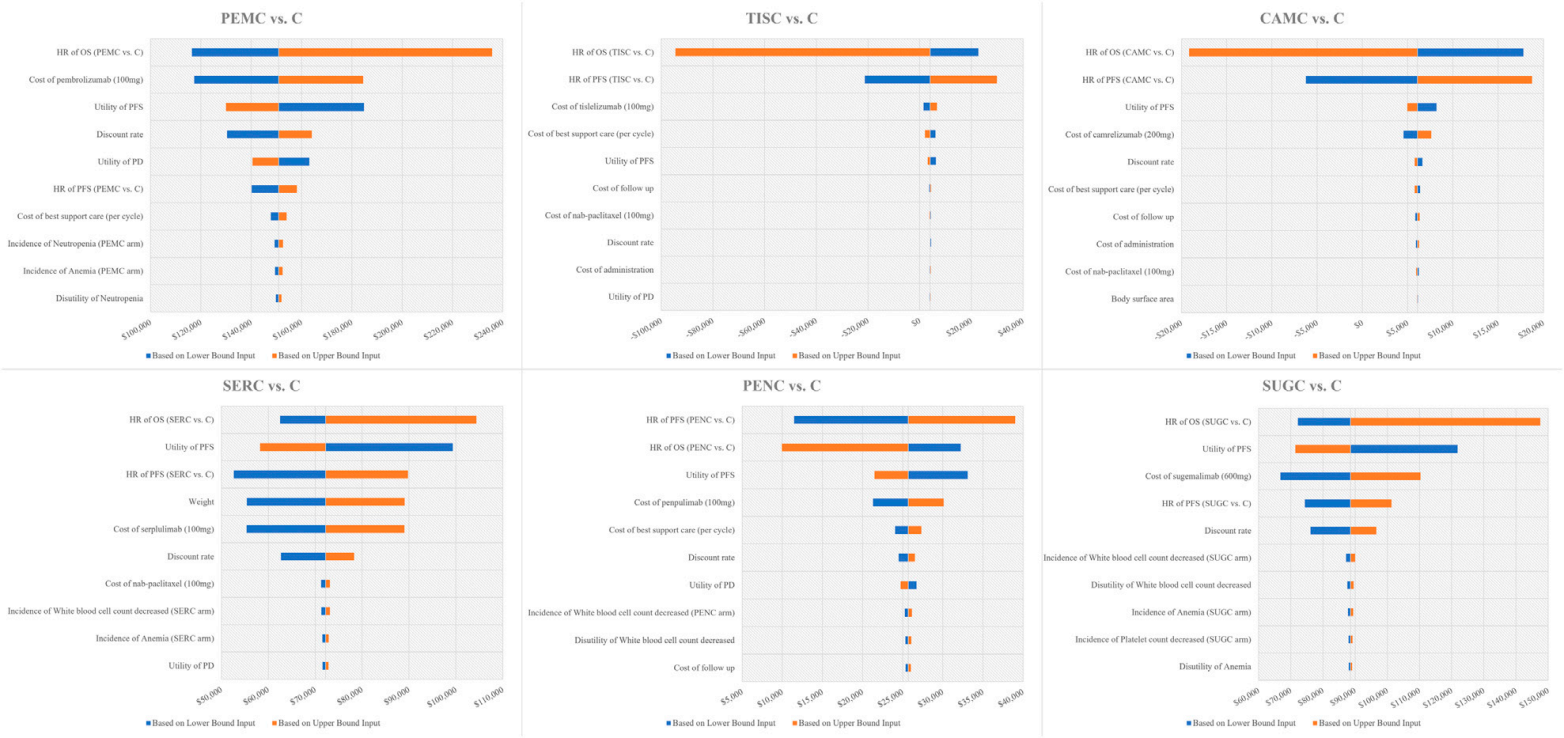
Tornado diagram of one-way sensitivity analysis results. Notes: The x--axis represents the incremental cost -utility ratio. Abbreviations: OS, overall survival; PFS, progression-free survival; HR, hazard ratio; C, chemotherapy; PEMC, pembrolizumab plus chemotherapy; TISC, tislelizumab plus chemotherapy; CAMC, camrelizumab plus chemotherapy; SERC, serplulimab plus chemotherapy, PENC, penpulimab plus chemotherapy; SUGC, sugemalimab plus chemotherapy.

In the DSA, tornado diagrams were generated to illustrate the impact of the input variations on the ICUR. The top 10 key inputs are presented in Figure 2 (Integrated tornado diagrams for six treatment comparisons: PEMC vs. C, TISC vs. C, CAMC vs. C, SERC vs. C, PENC vs. C, and SUGC vs. C)

For PEMC vs. C, the tornado diagram showed that the most sensitive variables driving ICUR fluctuations were the hazard ratio of OS (PEMC vs. C) and the cost of pembrolizumab (100 mg). The ICUR ranged from $116,437.6/QALY to $235,777.3/QALY, with these key inputs driving the ratio across a wide range. Variables such as the utility of PFS and the discount rate also exerted notable influences, though to a lesser extent than the top two factors.

Regarding TISC vs. C, the HR of OS (TISC vs. C) and HR of PFS (TISC vs. C) emerged as prominent sensitivity variables. The ICUR exhibited a unique range, from −$94,458.8/QALY to $29,874.0/QALY. Unlike PEMC vs. C, cost-related inputs such as the cost of tislelizumab (100 mg) and the cost of best supportive care had relatively smaller impacts.

For CAMC vs. C, similar to TISC vs. C in some aspects, the HR of OS (CAMC vs. C) and HR of PFS (CAMC vs. C) were the dominant sensitivity factors. The ICUR spanned from −$19,155.4/QALY to $18,759.6/QALY, and variables such as the utility of PFS and the cost of camrelizumab (200 mg) played secondary roles in shaping the ICUR’s fluctuation.

In the case of SERC vs. C, the HR of OS (SERC vs. C), utility of PFS, and HR of PFS (SERC vs. C) were identified as major sensitivity variables. The ICUR ranged from $52,611.1/QALY to $104,373.4/QALY, with the cost of SER (100 mg) and weight also influencing the results.

For PENC vs. C, the HR of PFS (PENC vs. C) and HR of OS (PENC vs. C) stood out as the most sensitive inputs. The ICUR fluctuated between $9,943.1/QALY and $39,021.5/QALY, and factors such as the utility of PFS and the cost of penpulimab (100 mg) contributed to the variability, though not as strongly as the HR values.

Regarding SUGC vs. C, the HR of OS (SUGC vs. C), utility of PFS, and cost of sugemalimab (600 mg) were the key sensitivity variables. The ICUR ranged from $66,797.7/QALY to $147,530.3/QALY. The HR of PFS (SUGC vs. C) and the discount rate also play crucial roles in driving the ICUR’s changes.

Across all six comparisons, it is worth noting that the WTP threshold was set at $40,334/QALY. For most treatment combinations (except TISC vs. C and CAMC vs. C, where ICURs fell below the WTP in some ranges), the upper bounds of ICURs exceeded this threshold, indicating that the cost-effectiveness profile could be sensitive to input fluctuations and highlighting the need for careful consideration of these key variables in decision-making.

#### 3.3.3 Probabilistic sensitivity analysis

PSA was performed with 1,000 Monte Carlo iterations, where all model inputs were simultaneously sampled from their respective probability distributions. The average results were generally in line with the base-case results (see Supplementary Table 5).

**FIGURE 3:**
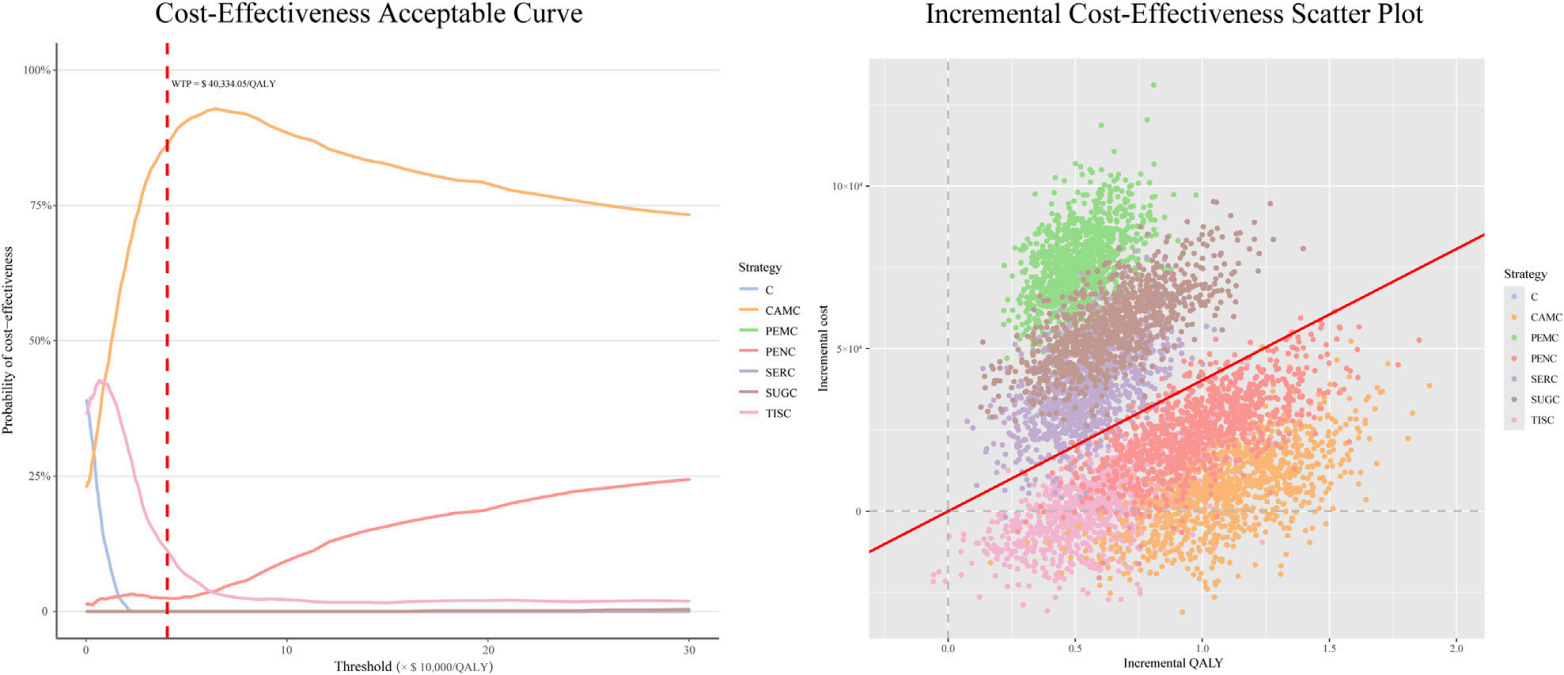
Probabilistic sensitivity analysis outputs for immune checkpoint inhibitor plus chemotherapy regimens vs. chemotherapy alone. Notes: For CEAC: the y-axis reflects the probability of a regimen being cost-effective across varying WTP thresholds (x-axis). The red dashed line marks the $40,334/QALY threshold. For the scatterplot: each dot represents one iteration result, with incremental costs (y-axis) and incremental QALYs (x-axis). The red line is the $40,334/QALY WTP threshold. Abbreviations: C, chemotherapy; PEMC, pembrolizumab plus chemotherapy; TISC, tislelizumab plus chemotherapy; CAMC, camrelizumab plus chemotherapy; SERC, serplulimab plus chemotherapy; PENC, penpulimab plus chemotherapy; SUGC, sugemalimab plus chemotherapy; QALY, quality-adjusted life-year; WTP, willingness to pay.

The CEAC (left panel of Figure 3) showed that the PEMC, SERC, and SUGC regimens had 0% probability of being cost-effective. Their CEAC curves remained near the 0% probability baseline at this threshold, indicating that they rarely achieve cost-effectiveness under typical WTP ($ 40,334/QALY) constraints.

The PENC regimen showed a 2.5% probability of being cost-effective, with its CEAC curve slightly above the baseline but still demonstrating limited cost-effectiveness potential. The TISC regimen reached an 11.4% cost-effectiveness probability, suggesting a low but non-negligible likelihood of cost-effectiveness at this threshold. The CAMC regimen achieved a cost-effectiveness probability of 86.1%. Its CEAC curve increases sharply and stabilizes at a high probability, underscoring its strong cost-effectiveness profile relative to other regimens across the WTP range.

Given that the ICURs of the three regimens—PENC, TISC, and CAMC—were all below the threshold of three times the *per capita* GDP, we further compared their cost-effectiveness by setting the WTP threshold at one times the *per capita* GDP and then calculated the probability of each regimen being cost-effective at this threshold. The results showed that the probability was 51.5% for CAMC, 39.5% for TISC, and 2.6% for PENC.

## 4 Discussion

NSCLC poses a severe threat to public health as it is one of the most fatal malignancies and imposes a substantial economic burden globally (Zhou et al., 2014). Research and development of novel anti-neoplastic drugs continuously generate more treatment options. Particularly, the advent of immunotherapy has dramatically changed the treatment landscape and become a mainstay of cancer therapy (Couzin-Frankel, 2013). New treatments, however, are usually associated with high costs. In the past few years, China’s National Healthcare Security Administration has been negotiating with pharmaceutical companies on the antineoplastic agents, resulting in a 30%–70% reduction in prices, including that of ICIs (National Healthcare Security Administration, 2022a; National Healthcare Security Administration, 2022b). Undoubtedly, it greatly reduces the financial burden of cancer patients. Given limited healthcare resources, pricing drugs based on their clinical value is essential and justified. Therefore, it is essential to consider economic factors along with clinical efficacy and safety when choosing therapies for patients. Our study evaluated the efficacy and economic outcomes of ICI plus chemotherapy regimens available in China for the treatment of squamous NSCLC from the perspective of the Chinese healthcare system.

In terms of survival benefit, six ICI plus chemotherapy regimens were superior to chemotherapy alone. Our NMA revealed that camrelizumab plus chemotherapy and penpulimab plus chemotherapy regimens yielded more OS survival benefits than other ICI plus chemotherapy regimens. In addition, the camrelizumab plus chemotherapy regimen produced more PFS survival benefits than other ICI plus chemotherapy regimens. Our economic analyses demonstrated that the camrelizumab plus chemotherapy regimen yielded more QALYs than other regimens, with penpulimab plus chemotherapy ranking second. Results from the base-case analyses revealed that the ICURs of three regimens, including tislelizumab plus chemotherapy, camrelizumab plus chemotherapy, and penpulimab plus chemotherapy regimens, were less than the WTP threshold of three times *per capita* GDP ($ 40,334/QALY). Among these, the tislelizumab plus chemotherapy regimen exhibited the lowest ICUR value, but the camrelizumab plus chemotherapy regimen demonstrated a comparable ICUR while providing more QALYs.

The pembrolizumab plus chemotherapy regimen had the lowest QALYs and highest costs, which makes it an inferior strategy among the six ICI plus chemotherapy regimens. Both the sugemalimab plus chemotherapy and serplulimab plus chemotherapy regimens showed poor performance in cost-effectiveness. DSA indicated that the HR of the ICI plus chemotherapy regimen versus chemotherapy was the key input driving ICUR fluctuations. The outputs of PSA also confirmed the robustness of base-case findings. When the WTP threshold was adjusted to $13,445/QALY, camrelizumab plus chemotherapy still had the highest cost-effectiveness probability (51.5%), followed by tislelizumab plus chemotherapy (39.5%) and penpulimab plus chemotherapy (2.6%). The observed differences in outcomes among the six regimens are notable, and we analyzed the potential contributing factors related to both clinical trial design and the mechanistic or structural characteristics of the drugs. Although all six are PD-1/PD-L1-targeting ICIs, subtle differences in their mechanistic and structural properties may explain the variations in efficacy. A summary of these distinguishing features for each agent is provided in Supplementary Table 6. Additionally, minor differences in trial design across studies may have influenced the comparative results. For instance, some trials enrolled a higher proportion of metastatic patients, who typically have poorer prognoses than those with locally advanced disease. This variation in the patient mix could contribute to somewhat less favorable outcomes in the trials with larger metastatic cohorts. These mechanistic differences and trial design nuances collectively help explain the divergent efficacy profiles observed across the six treatment regimens. Such factors also underscore the importance of contextualizing our findings within the specific design and the patient population of each trial as they reflect the inherent complexities of indirect treatment comparisons in advanced oncology research.

To our knowledge, this study is the first to synthesize the most recent survival data for six available ICI plus chemotherapy regimens and establish connections for indirect comparisons through an NMA. It also conducted an economic evaluation of the six regimens to identify the preferred first-line treatment for squamous NSCLC. Although the proportion of squamous NSCLC is not as high as that of non-squamous NSCLC, it is still a major clinical challenge. Notably, among the six regimens, tislelizumab plus chemotherapy, camrelizumab plus chemotherapy, and penpulimab plus chemotherapy stand out in terms of cost savings and survival benefits. Accordingly, this study could assist Chinese policymakers and clinical guideline developers in determining which regimen should be recommended as the top-priority option for the treatment of squamous NSCLC.

There are several limitations to the current study. First, although our analysis was designed from a Chinese perspective, our NMA did not restrict the nationality or ethnicity of patients in the original trials. This creates uncertainty about how the results generalize to the specific Chinese population. Additionally, to build connected comparison paths for the NMA, we overlooked minor differences in target patient characteristics across trials, such as variations in comorbidities or prior treatment histories. These patient-related inconsistencies, along with other trial-specific factors, contribute to potential heterogeneity. We used a fixed-effects model for the NMA, a choice justified by the included PD-1/PD-L1 inhibitors’ shared mechanism of action and alignment with common NMA practices for closely related oncology regimens. However, unmeasured heterogeneity may persist, including that from differences in chemotherapy backbones or dosing schedules across trials. Second, although the CSCO guidelines recommended sintilimab for the treatment of squamous NSCLC, it was not included in this analysis due to immature OS data from the ORIENT-12 trial (Zhou et al., 2021). Third, the absence of closed loops in the network prevented consistency testing, which may introduce bias. Fourth, in order to estimate long-term survival benefit, a range of parametric survival functions was fitted to the pooled IPD from the chemotherapy arm. The pseudo-IPD was generated using an algorithm proposed by Guyot et al. (2012) rather than real data from individual patients. Guyot’s algorithm is often applied in economic analyses and has been proven to outperform other methods (Saluja et al., 2019). In addition, the survival data of ICI plus chemotherapy regimens were generated through the HR values of ICI plus chemotherapy against chemotherapy. However, it is inevitable that algorithms and modeling techniques would cause uncertainty. Fifth, our model assumed docetaxel as the uniform subsequent-line therapy for all regimens after progression, which may not reflect real-world clinical practice. In reality, post-progression treatments vary widely, including immune rechallenge, antiangiogenic drugs, or other targeted therapies. This homogenization of second-line therapy may underestimate the cost-effectiveness of regimens that are more likely to be followed by effective subsequent treatments in clinical practice. Finally, a notable limitation is our inability to conduct subgroup analyses, particularly stratified by PD-L1 expression status. PD-L1 expression is a well-recognized predictive biomarker for ICI efficacy. The six included clinical trials primarily reported outcomes for the overall population group, which encompasses patients with varying PD-L1 expression levels (both <1% and ≥1%). Although available subgroup data from some trials indicated differential benefit magnitudes by PD-L1 status, critical OS data for PD-L1 subgroups were incompletely reported in trials such as the ASTRUM-004, GEMSTONE-302, and RATIONALE-403 trials. This data gap, combined with the inconsistency of subgroup characteristics across the included clinical trials, precluded detailed subgroup-specific economic evaluations, including analyses of how PD-L1 expression influences the cost-effectiveness of ICI plus chemotherapy regimens. These represent important directions for future research with more granular patient-level data and standardized subgroup reporting.

Our reported comparative effectiveness estimates reflect the average treatment effects rather than context-specific outcomes, so conclusions should be interpreted cautiously. Clinical decisions for Chinese patients should also consider the unique demographic and clinical characteristics of the local population. Future studies based on individual patient data from Chinese cohorts could better address this heterogeneity and refine the applicability of our findings.

## 5 Conclusion

The network meta-analyses revealed that the camrelizumab plus chemotherapy regimen and penpulimab plus chemotherapy regimens yielded more OS survival benefits than other ICI plus chemotherapy regimens. In addition, the camrelizumab plus chemotherapy regimen produced more PFS survival benefits than other ICI plus chemotherapy regimens. Economic evaluations demonstrated that among the six ICI plus chemotherapy regimens evaluated, tislelizumab plus chemotherapy demonstrated the lowest ICUR, followed by the camrelizumab plus chemotherapy regimen. However, with a threshold of $13,445/QALY or $40,334/QALY, camrelizumab plus chemotherapy provided greater QALY benefits than tislelizumab plus chemotherapy. Thus, camrelizumab plus chemotherapy is recommended as the preferred first-line treatment for advanced squamous non-small cell lung cancer in this context.

## Data Availability

The original contributions presented in the study are included in the article/Supplementary Material, further inquiries can be directed to the corresponding author.
