# Supplementary material for: Immune checkpoint blockers plus chemotherapy as the first-line treatment for advanced or metastatic squamous non-small-cell lung carcinoma: a network meta-analysis and economic evaluation
Source: Front Pharmacol. 2025 Oct 21;16:1669965. doi: 10.3389/fphar.2025.1669965 (PMC12582988; doi:10.3389/fphar.2025.1669965)
Supplement: Supplementary file 1 [file Supplementaryfile1.docx]

Supplementary Information

[Supplementary Table 1. PRISMA NMA Checklist of Items to Include When Reporting A Systematic Review Involving a Network Meta-analysis 2](#_Toc208927031)

[Supplementary Table 2. Inclusion and exclusion criteria of the network meta-analysis 4](#_Toc208927032)

[Supplementary Table 3. Characteristics of the clinical trials included in the network Meta-analysis 4](#_Toc208927033)

[Supplementary Table 4. The scale and shape parameters of the projected curve of the chemotherapy arm 5](#_Toc208927034)

[Supplementary Table 5. Average Results of the Probabilistic Sensitivity Analysis 5](#_Toc208927035)

[Supplementary Table 6: Mechanistic and Structural Characteristics of the Investigated Immune Checkpoint Inhibitors (ICIs) 5](#_Toc208927036)

[Supplementary content 1. Retrieval strategies for PubMed and Cochrane Library 6](#_Toc208927037)

[Supplementary Figure 1. Flowchart of study selection. 8](#_Toc208927038)

[Supplementary Figure 2. The network plot of all trials. 9](#_Toc208927039)

[Supplementary Figure 3. Traffic-light plot: risk of bias summary. 9](#_Toc208927040)

[Supplementary Figure 4. The reconstructed Kaplan-Meier OS and PFS curves based on the pooled time-to-event data of chemotherapy regimen. 10](#_Toc208927041)

[Supplementary Figure 5. Diagram of projected PFS and OS fit curves based on the hazard ratio of network meta-analysis. 11](#_Toc208927042)

[Supplemental Figure 6. Proportion of patients at each time point. 11](#_Toc208927043)

[Reference: 11](#_Toc208927044)

Supplementary Table 1. PRISMA NMA Checklist of Items to Include When Reporting A Systematic Review Involving a Network Meta-analysis

| **Section/Topic** | **Item #** | **Checklist Item** | **Article section** |
| --- | --- | --- | --- |
| **TITLE** |  |  |  |
| Title | 1 | Identify the report as a systematic review *incorporating a network meta-analysis (or related form of meta-analysis).* | Title |
| **ABSTRACT** |  |  |  |
| Structured summary | 2 | Provide a structured summary including, as applicable:  **Background:** main objectives  **Methods:** data sources; study eligibility criteria, participants, and interventions; study appraisal; and *synthesis methods, such as network meta-analysis.*  **Results:** number of studies and participants identified; summary estimates with corresponding confidence/credible intervals; *treatment rankings may also be discussed. Authors may choose to summarize pairwise comparisons against a chosen treatment included in their analyses for brevity.*  **Discussion/Conclusions:** limitations; conclusions and implications of findings.  **Other:** primary source of funding; systematic review registration number with registry name. | Abstract |
| **INTRODUCTION** |  |  |  |
| Rationale | 3 | Describe the rationale for the review in the context of what is already known*, including mention of why a network meta-analysis has been conducted.* | Introduction |
| Objectives | 4 | Provide an explicit statement of questions being addressed, with reference to participants, interventions, comparisons, outcomes, and study design (PICOS). | Introduction |
|  |  |  |  |
| **METHODS** |  |  |  |
| Protocol and registration | 5 | Indicate whether a review protocol exists and if and where it can be accessed (e.g., Web address); and, if available, provide registration information, including registration number. | Not applicable |
| Eligibility criteria | 6 | Specify study characteristics (e.g., PICOS, length of follow-up) and report characteristics (e.g., years considered, language, publication status) used as criteria for eligibility, giving rationale. *Clearly describe eligible treatments included in the treatment network, and note whether any have been clustered or merged into the same node (with justification).* | Supplementary Table 2 |
| Information sources | 7 | Describe all information sources (e.g., databases with dates of coverage, contact with study authors to identify additional studies) in the search and date last searched. | Materials and Methods |
| Search | 8 | Present full electronic search strategy for at least one database, including any limits used, such that it could be repeated. | Supplementary content 1 |
| Study selection | 9 | State the process for selecting studies (i.e., screening, eligibility, included in systematic review, and, if applicable, included in the meta-analysis). | Materials and Methods |
| Data collection process | 10 | Describe method of data extraction from reports (e.g., piloted forms, independently, in duplicate) and any processes for obtaining and confirming data from investigators. | Materials and Methods |
| Data items | 11 | List and define all variables for which data were sought (e.g., PICOS, funding sources) and any assumptions and simplifications made. | Materials and Methods |
| **Geometry of the network** | **S1** | Describe methods used to explore the geometry of the treatment network under study and potential biases related to it. This should include how the evidence base has been graphically summarized for presentation, and what characteristics were compiled and used to describe the evidence base to readers. | Materials and Methods |
| Risk of bias within individual studies | 12 | Describe methods used for assessing risk of bias of individual studies (including specification of whether this was done at the study or outcome level), and how this information is to be used in any data synthesis. | Materials and Methods |
| Summary measures | 13 | State the principal summary measures (e.g., risk ratio, difference in means). *Also describe the use of additional summary measures assessed, such as treatment rankings and surface under the cumulative ranking curve (SUCRA) values, as well as modified approaches used to present summary findings from meta-analyses.* | Materials and Methods |
| Planned methods of analysis | 14 | Describe the methods of handling data and combining results of studies for each network meta-analysis. This should include, but not be limited to:   - *Handling of multi-arm trials;* - *Selection of variance structure;* - *Selection of prior distributions in Bayesian analyses; and* - *Assessment of model fit.* | Materials and Methods |
| **Assessment of Inconsistency** | **S2** | Describe the statistical methods used to evaluate the agreement of direct and indirect evidence in the treatment network(s) studied. Describe efforts taken to address its presence when found. | Materials and Methods |
| Risk of bias across studies | 15 | Specify any assessment of risk of bias that may affect the cumulative evidence (e.g., publication bias, selective reporting within studies). | Not applicable |
| Additional analyses | 16 | Describe methods of additional analyses if done, indicating which were pre-specified. This may include, but not be limited to, the following:   - Sensitivity or subgroup analyses; - Meta-regression analyses; - *Alternative formulations of the treatment network; and* - *Use of alternative prior distributions for Bayesian analyses (if applicable).* | Not applicable |
| **RESULTS†** |  |  |  |
| Study selection | 17 | Give numbers of studies screened, assessed for eligibility, and included in the review, with reasons for exclusions at each stage, ideally with a flow diagram. | Supplementary Figure 1 |
| **Presentation of network structure** | **S3** | Provide a network graph of the included studies to enable visualization of the geometry of the treatment network. | Supplementary Figure 2 |
| **Summary of network geometry** | **S4** | Provide a brief overview of characteristics of the treatment network. This may include commentary on the abundance of trials and randomized patients for the different interventions and pairwise comparisons in the network, gaps of evidence in the treatment network, and potential biases reflected by the network structure. | Not applicable |
| Study characteristics | 18 | For each study, present characteristics for which data were extracted (e.g., study size, PICOS, follow-up period) and provide the citations. | Supplementary Table 3 |
| Risk of bias within studies | 19 | Present data on risk of bias of each study and, if available, any outcome level assessment. | Supplementary Figure 3 |
| Results of individual studies | 20 | For all outcomes considered (benefits or harms), present, for each study: 1) simple summary data for each intervention group, and 2) effect estimates and confidence intervals. *Modified approaches may be needed to deal with information from larger networks.* | 23 |
| Synthesis of results | 21 | Present results of each meta-analysis done, including confidence/credible intervals. *In larger networks, authors may focus on comparisons versus a particular comparator (e.g. placebo or standard care), with full findings presented in an appendix. League tables and forest plots may be considered to summarize pairwise comparisons.* If additional summary measures were explored (such as treatment rankings), these should also be presented. | Table 2 |
| **Exploration for inconsistency** | **S5** | Describe results from investigations of inconsistency. This may include such information as measures of model fit to compare consistency and inconsistency models, *P* values from statistical tests, or summary of inconsistency estimates from different parts of the treatment network. | Not applicable |
| Risk of bias across studies | 22 | Present results of any assessment of risk of bias across studies for the evidence base being studied. | Not applicable |
| Results of additional analyses | 23 | Give results of additional analyses, if done (e.g., sensitivity or subgroup analyses, meta-regression analyses*, alternative network geometries studied, alternative choice of prior distributions for Bayesian analyses,* and so forth). | Not applicable |
| **DISCUSSION** |  |  |  |
| Summary of evidence | 24 | Summarize the main findings, including the strength of evidence for each main outcome; consider their relevance to key groups (e.g., healthcare providers, users, and policy-makers). | Discussion |
| Limitations | 25 | Discuss limitations at study and outcome level (e.g., risk of bias), and at review level (e.g., incomplete retrieval of identified research, reporting bias). *Comment on the validity of the assumptions, such as transitivity and consistency. Comment on any concerns regarding network geometry (e.g., avoidance of certain comparisons).* | Discussion |
| Conclusions | 26 | Provide a general interpretation of the results in the context of other evidence, and implications for future research. | Conclusion |
| **FUNDING** |  |  |  |
| Funding | 27 | Describe sources of funding for the systematic review and other support (e.g., supply of data); role of funders for the systematic review. This should also include information regarding whether funding has been received from manufacturers of treatments in the network and/or whether some of the authors are content experts with professional conflicts of interest that could affect use of treatments in the network. | Declarations |

PICOS = population, intervention, comparators, outcomes, study design.

* Text in italics indicateS wording specific to reporting of network meta-analyses that has been added to guidance from the PRISMA statement.

† Authors may wish to plan for use of appendices to present all relevant information in full detail for items in this section.

Supplementary Table 2. Inclusion and exclusion criteria of the network meta-analysis

| Characteristics | Inclusion criteria | Exclusion criteria |
| --- | --- | --- |
| Population | Adult patients with unresectable metastatic or advanced squamous NSCLC. | Age younger than 18 years;  Healthy subjects or non-squamous NSCLC patients. |
| Intervention | CSCO guideline recommended ICI plus chemotherapy schedules;  ICI plus chemotherapy regimen approved by NMPA for first-line treatment in China. | Interventions not for first-line regimen;  ICI monotherapy regimen;  ICIs that have not been approved in China. |
| Comparator | Chemotherapy alone. | Non-pharmaceutical treatments;  Placebo. |
| Outcomes | PFS and OS data;  PFS and OS HR of intervention versus control. | Non-survival outcomes;  Only the PFS or OS data;  Immature data. |
| Study design | Clinical trial;  Phase Ⅲ. | Non-clinical trial;  Non-phase Ⅲ trial. |

**Abbreviation:** ***PFS***, Progression-free survival; ***OS***, Overall survival; ***CSCO***, Chinese society of clinical oncology; ***HR***, hazard ratio; ***ICI***, Immune checkpoint inhibitor; ***NSCLC***, non-small cell lung carcinoma; ***NMPA***, National Medical Products Administration.

Supplementary Table 3. Characteristics of the clinical trials included in the network Meta-analysis

| Study | Author | Year | Sample size | Patient | Intervention | Comparator | median follow-up, months | HR of PFS (95% CI) | HR of OS (95% CI) |
| --- | --- | --- | --- | --- | --- | --- | --- | --- | --- |
| KEYNOTE-407 | Novello S. et al | 2023 | 559 | NSCLC | Pembrolizumab plus chemotherapy | Chemotherapy | 56.9 | 0.62(0.52 – 0.74) | 0.71 (0.59 – 0.85) |
| RATIONALE-307 | Wang J. et al | 2024 | 241 | NSCLC | Tislelizumab plus chemotherapy | Chemotherapy | 16.7 | 0.45 (0.33 –0.62) | 0.69 (0.50 –0.95) |
| CameL-sq | Ren S. et al | 2021 | 389 | NSCLC | Camrelizumab Plus Chemotherapy | Chemotherapy | 13.5 | 0.37 (0.29 – 0.47) | 0.55 (0.40 – 0.75) |
| ASTRUM-004 | Zhou C. et al | 2024 | 537 | NSCLC | Serplulimab Plus Chemotherapy | Chemotherapy | 8.9 | 0.53 (0.42 – 0.67) | 0.73 (0.58 – 0.93) |
| AK105-302 | Zhong H. et al | 2024 | 350 | NSCLC | Penpulimab plus chemotherapy | Chemotherapy | 24.7 | 0.43 (0.33 – 0.56) | 0.55 (0.40 – 0.75) |
| GEMSTONE-302 | Zhou C. et al | 2022 | 479 | NSCLC | Sugemalimab plus chemotherapy | Chemotherapy | 17.8 | 0.48 (0.39 – 0.60) | 0.67 (0.5 – 0.9) |

**Abbreviation:** ***PFS***, Progression-free survival; ***OS***, Overall survival; ***HR***, hazard ratio; ***CI***, Confidence interval; ***NSCLC***, Squamous non-small cell lung carcinoma.

Supplementary Table 4. The scale and shape parameters of the projected curve of the chemotherapy arm

| **Distributions** | **Parameters** | **PFS** | | | | **OS** | | | |
| --- | --- | --- | --- | --- | --- | --- | --- | --- | --- |
|  |  | **Estimate** | **L95%** | **U95%** | **Standard Error** | **Estimate** | **L95%** | **U95%** | **Standard Error** |
| **Log-logistic** | Shape | 2.0766 | 1.9663 | 2.193 | 0.0578 | 1.5621 | 1.4688 | 1.6613 | 0.0491 |
|  | Scale | 7.1862 | 6.8354 | 7.555 | 0.1835 | 23.0596 | 21.511 | 24.7197 | 0.8179 |
|  | AIC | 5938.948 | | | | 6437.837 | | | |
|  | BIC | 5948.974 | | | | 6447.864 | | | |
| **Weibull** | Shape | 1.1032 | 1.0552 | 1.1534 | 0.0251 | 1.1498 | 1.0839 | 1.2198 | 0.0346 |
|  | Scale | 11.298 | 10.6404 | 11.9962 | 0.3457 | 34.6036 | 32.4543 | 36.8952 | 1.1321 |
|  | AIC | 6248.122 | | | | 6481.541 | | | |
|  | BIC | 6258.148 | | | | 6491.567 | | | |
| **Exp** | Rate | 0.09078 | 0.0851 | 0.09684 | 0.00299 | 0.02836 | 0.02635 | 0.03052 | 0.00106 |
|  | AIC | 6263.555 | | | | 6499.556 | | | |
|  | BIC | 6268.568 | | | | 6504.569 | | | |
| **Lognormal** | Meanlog | 1.983 | 1.928 | 2.038 | 0.028 | 3.1393 | 3.0644 | 3.2142 | 0.0382 |
|  | Sdlog | 0.894 | 0.854 | 0.936 | 0.021 | 1.1453 | 1.0847 | 1.2092 | 0.0318 |
|  | AIC | 6003.107 | | | | 6458.982 | | | |
|  | BIC | 6013.133 | | | | 6469.008 | | | |
| **Gompertz** | Shape | -0.01679 | -0.02274 | -0.01083 | 0.00304 | 0.000636 | -0.003902 | 0.005175 | 0.002316 |
|  | Rate | 0.10628 | 0.09802 | 0.11523 | 0.00439 | 0.028033 | 0.02511 | 0.031297 | 0.001575 |
|  | AIC | 6228.115 | | | | 6501.482 | | | |
|  | BIC | 6238.141 | | | | 6511.508 | | | |
| **Abbreviations:** ***L95%*** Lower 95% confidence limit, ***U95%*** Upper 95% confidence limit. | | | | | | | | | |

Supplementary Table 5. Average Results of the Probabilistic Sensitivity Analysis

| **Regimen** | Chemotherapy | PEMC | TISC | CAMC | SERC | PENC | SUGC |
| --- | --- | --- | --- | --- | --- | --- | --- |
| **Total cost** | $40,644.5 | $117,556.5 | $42,751.8 | $47,259.5 | $78,197.5 | $65,652.3 | $97,378.1 |
| Difference (vs. Chemotherapy) | - | $76,912.0 | $2,107.3 | $6,615.0 | $37,553.0 | $25,007.8 | $56,733.6 |
| **Total QALY** | 1.256 | 1.775 | 1.86 | 2.367 | 1.798 | 2.245 | 1.911 |
| Difference (vs. Chemotherapy) | - | 0.519 | 0.604 | 1.111 | 0.542 | 0.989 | 0.655 |
| **ICUR($/QALY)** | - | $148,192.68 | $3,488.91 | $5,954.10 | $69,285.98 | $25,285.95 | $86,616.18 |
| **Abbreviations:** ***PEMC*** *pembrolizumab plus chemotherapy,* ***TISC*** *tislelizumab plus chemotherapy,* ***CAMC*** *camrelizumab plus chemotherapy,* ***SERC*** *serplulimab plus chemotherapy,* ***PENC*** *penpulimab plus chemotherapy,* ***SUGC*** *sugemalimab plus chemotherapy,* ***C*** *chemotherapy,* ***PFS*** *progression-free survival,* ***OS*** *overall survival*, ***QALY*** quality-adjusted life-year, ***LY*** life-year, ***ICUR*** incremental cost-utility ratio. | | | | | | | |

Supplementary Table 6: Mechanistic and Structural Characteristics of the Investigated Immune Checkpoint Inhibitors (ICIs)

| ICI Name | Target | Antibody Isotype | Fc Region Modification | Binding Epitope on Target | Mechanism of Action | References |
| --- | --- | --- | --- | --- | --- | --- |
| Pembrolizumab | PD-1 | IgG4 | Hinge modification to prevent IgG4 half-molecule exchange | Multiple residues on PD-1 loops overlapping with PD-L1 interface | Blocks PD-1/PD-L1 and PD-1/PD-L2 binding, restoring T-cell activation | (Yang et al., 2015; Fessas et al., 2017; Wang et al., 2019; Hutchins et al., 2020; Gordeev et al., 2024) |
| Tislelizumab | PD-1 | IgG4 | Triple Fc mutations to abolish Fcγreceptor binding | CC′ loop region on PD-1 β-sheet face | Blocks PD-1/PD-L1 and PD-1/PD-L2 binding; reduced Fc-mediated T-cell clearance | (Zhang et al., 2022) |
| Camrelizumab | PD-1 | IgG4 | Some Fcγreceptor binding retained | Epitope not fully mapped; overlaps with PD-L1/PD-L2 binding interface | Blocks PD-1/PD-L1 and PD-1/PD-L2 interactions, enhancing T-cell immune responses | (Xu and Sun, 2022) |
| Serplulimab | PD-1 | IgG4 | Not clearly reported | Overlaps with PD-L1 binding interface | Blocks PD-1/PD-L1 interaction and induces PD-1 receptor internalization, sustaining T-cell activity | (Jiang et al., 2023; Ho et al., 2024) |
| Penpulimab | PD-1 | IgG1 | Fc engineered to eliminate Fcγreceptor binding | N58 glycosylation site on PD-1 BC loop | Blocks PD-1/PD-L1 and PD-1/PD-L2; Fc silencing reduces irAEs and improves efficacy | (Huang et al., 2022) |
| Sugemalimab | PD-L1 | IgG4 | Retain Fcγ receptor I binding ability | PD-L1 (blocks PD-1 and CD80 binding) | Blocks interaction of PD-L1 with PD-1 and B7.1, restoring T-cell activity similar to other PD-1 mAbs | (Zhou et al., 2022) |

Supplementary content 1. Retrieval strategies for PubMed and Cochrane Library

January 1, 2000 to December 31, 2024.

PubMed: 2,512 results

#1 (pembrolizumab[Title/Abstract]) OR (SCH-900475[Title/Abstract]) OR (lambrolizumab[Title/Abstract]) OR (MK-3475[Title/Abstract]) OR (Keytruda[Title/Abstract]) ------ 11,101 results

#2 (tislelizumab[Title/Abstract]) OR (BGB-A317[Title/Abstract]) OR (tislelizumab-jsgr[Title/Abstract]) OR (JHL-2108 [Title/Abstract]) OR (JHL2108[Title/Abstract]) OR (tevimbra[Title/Abstract]) ------ 529 results

#3 (camrelizumab[Title/Abstract]) OR (carrelizumab[Title/Abstract]) OR (SHR-1210[Title/Abstract]) OR (SHR 1210[Title/Abstract]) ------ 865 results

#4 (sintilimab[Title/Abstract]) OR (IBI 308[Title/Abstract]) OR (IBI308[Title/Abstract]) OR (IBI-308[Title/Abstract]) ------ 775 results

#5 (penpulimab[Title/Abstract]) ------ 31 results

#6 (serplulimab[Title/Abstract]) ------ 72 results

#7 (sugemalimab[Title/Abstract]) ------ 42 results

#8 (non-small cell lung carcinoma[Title/Abstract]) OR (Carcinoma, Non Small Cell Lung[Title/Abstract]) OR (Carcinomas, Non-Small-Cell Lung[Title/Abstract]) OR (Lung Carcinoma, Non-Small-Cell[Title/Abstract]) OR (Lung Carcinomas, Non-Small-Cell[Title/Abstract]) OR (Non-Small-Cell Lung Carcinomas[Title/Abstract]) OR (Non-Small Cell Lung Carcinoma[Title/Abstract]) OR (Non-Small-Cell Lung Carcinoma[Title/Abstract]) OR (Non Small Cell Lung Carcinoma[Title/Abstract]) OR (Nonsmall Cell Lung Cancer[Title/Abstract]) OR (Carcinoma, Non-Small Cell Lung[Title/Abstract]) OR (Non-Small Cell Lung Cancer[Title/Abstract]) OR (NSCLC[Title/Abstract]) ------ 102,772 results

#9 ("2000/01/01"[Date - Publication] : "2024/12/31"[Date - Publication]) ------ 24,652,871 results

(#1 OR #2 OR #3 OR #4 OR #5 OR #6 OR #7) AND (#8) AND (#9) ------ 2,512 results

Cochrane database: 380 results

January 1, 2000 to December 31, 2024.

#1 (pembrolizumab):ti,ab,kw OR (SCH-900475):ti,ab,kw OR (lambrolizumab):ti,ab,kw OR (MK-3475):ti,ab,kw OR (Keytruda):ti,ab,kw ------ 3,971 results

#2 (tislelizumab):ti,ab,kw OR (BGB-A317):ti,ab,kw OR (tislelizumab-jsgr):ti,ab,kw OR (JHL-2108):ti,ab,kw OR (tevimbra):ti,ab,kw ------ 393 results

#3 (camrelizumab):ti,ab,kw OR (carrelizumab):ti,ab,kw OR (SHR-1210):ti,ab,kw OR (SHR1210):ti,ab,kw ------ 392 results

#4 (sintilimab):ti,ab,kw OR (IBI 308):ti,ab,kw OR (IBI308):ti,ab,kw OR (IBI-308):ti,ab,kw ------ 287 results

#5 (penpulimab):ti,ab,kw ------ 43 results

#6 (Serplulimab):ti,ab,kw------ 57 results

#7 (sugemalimab):ti,ab,kw ------ 24 results

#8 MeSH descriptor: [Carcinoma, Non-Small-Cell Lung] explode all trees ------ 6,807 results

#9 (#1 OR #2 OR #3 OR #4 OR #5 OR #6 OR #7) AND (#8) with Cochrane Library publication date from Jan 2000 to Jan 2024 ------ 380 results


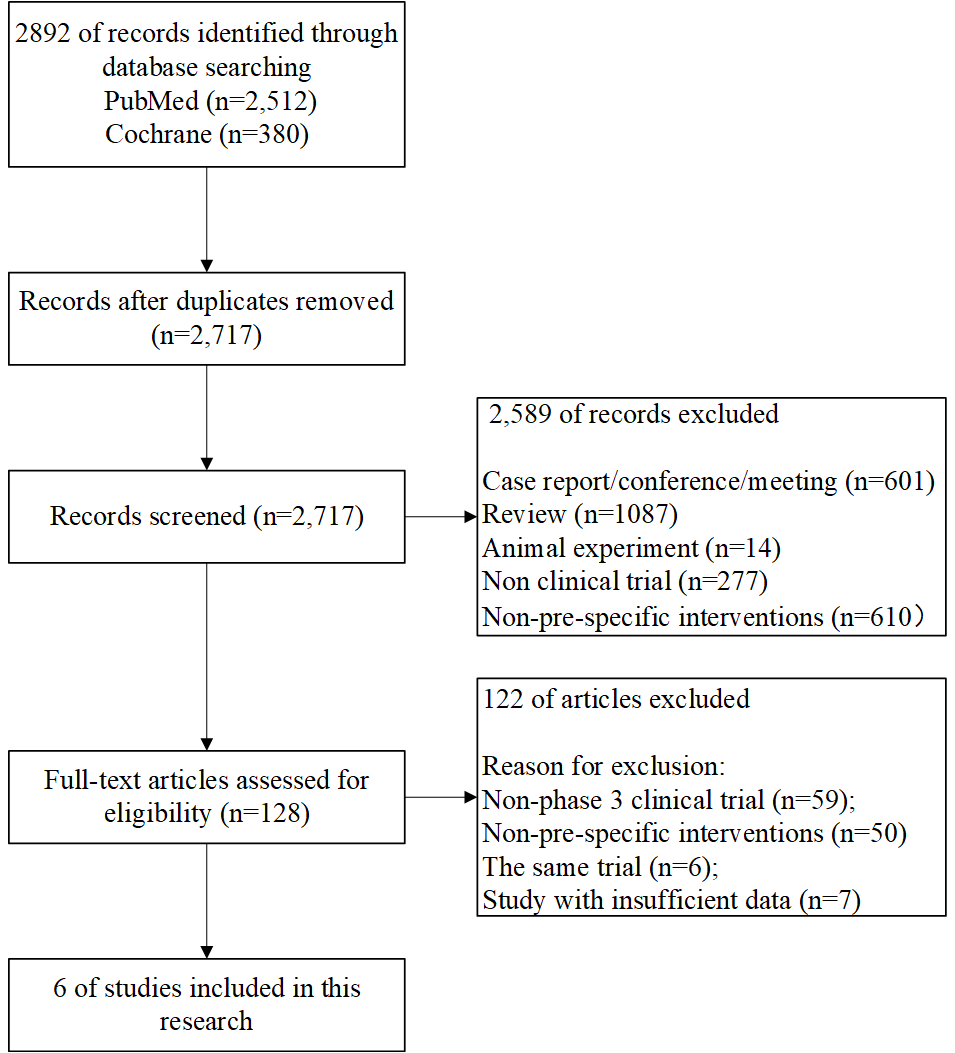


Supplementary Figure 1. Flowchart of study selection.


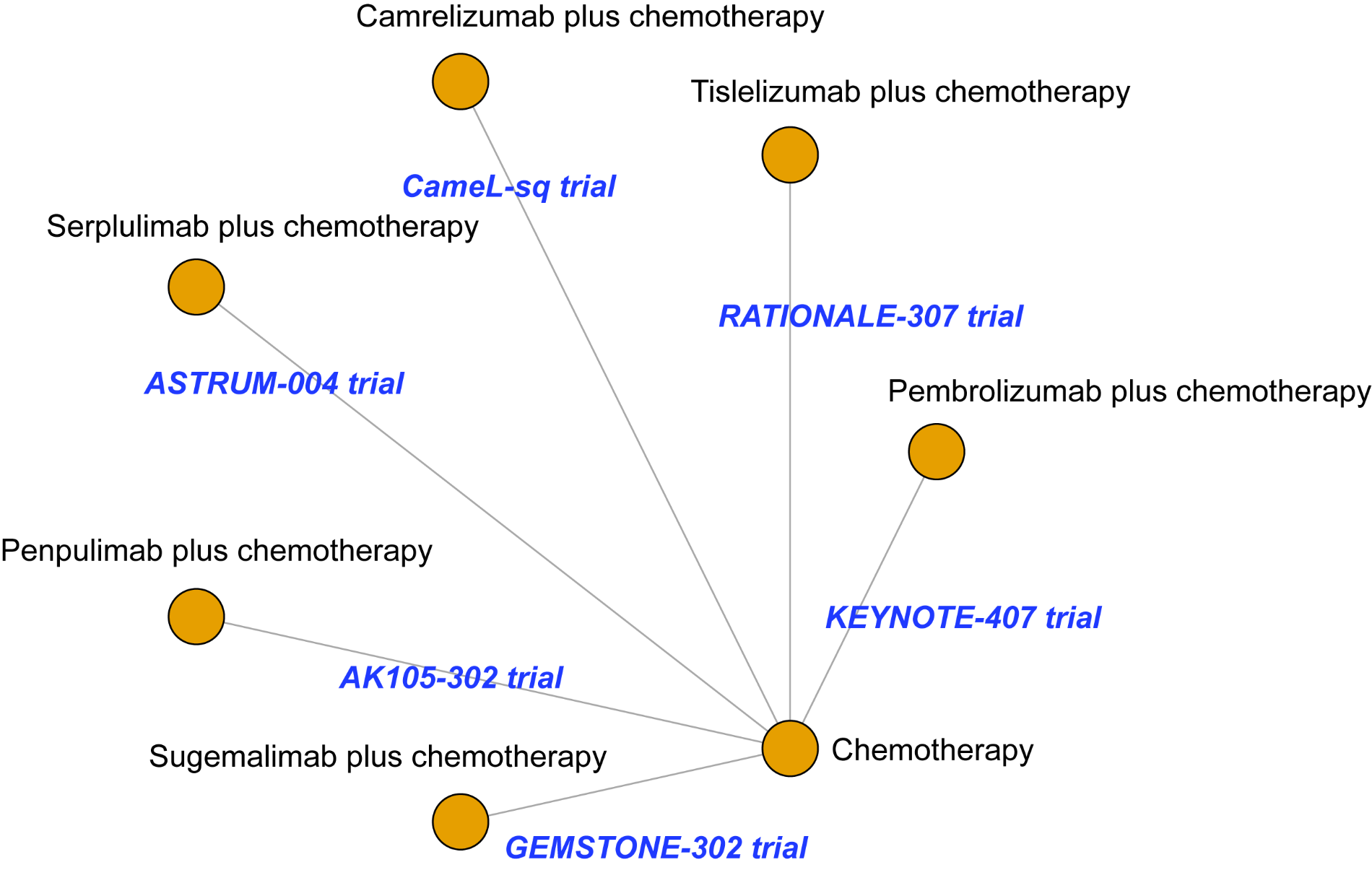


Supplementary Figure 2. The network plot of all trials.


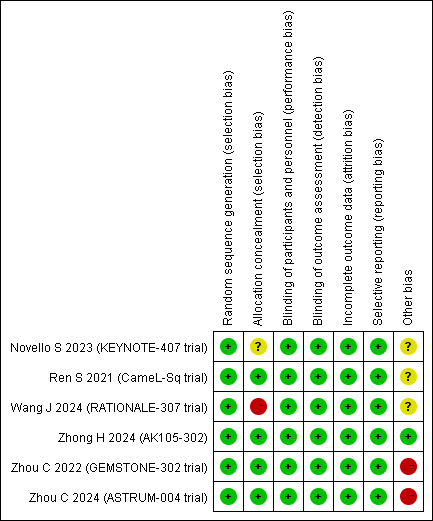


Supplementary Figure 3. Traffic-light plot: risk of bias summary.


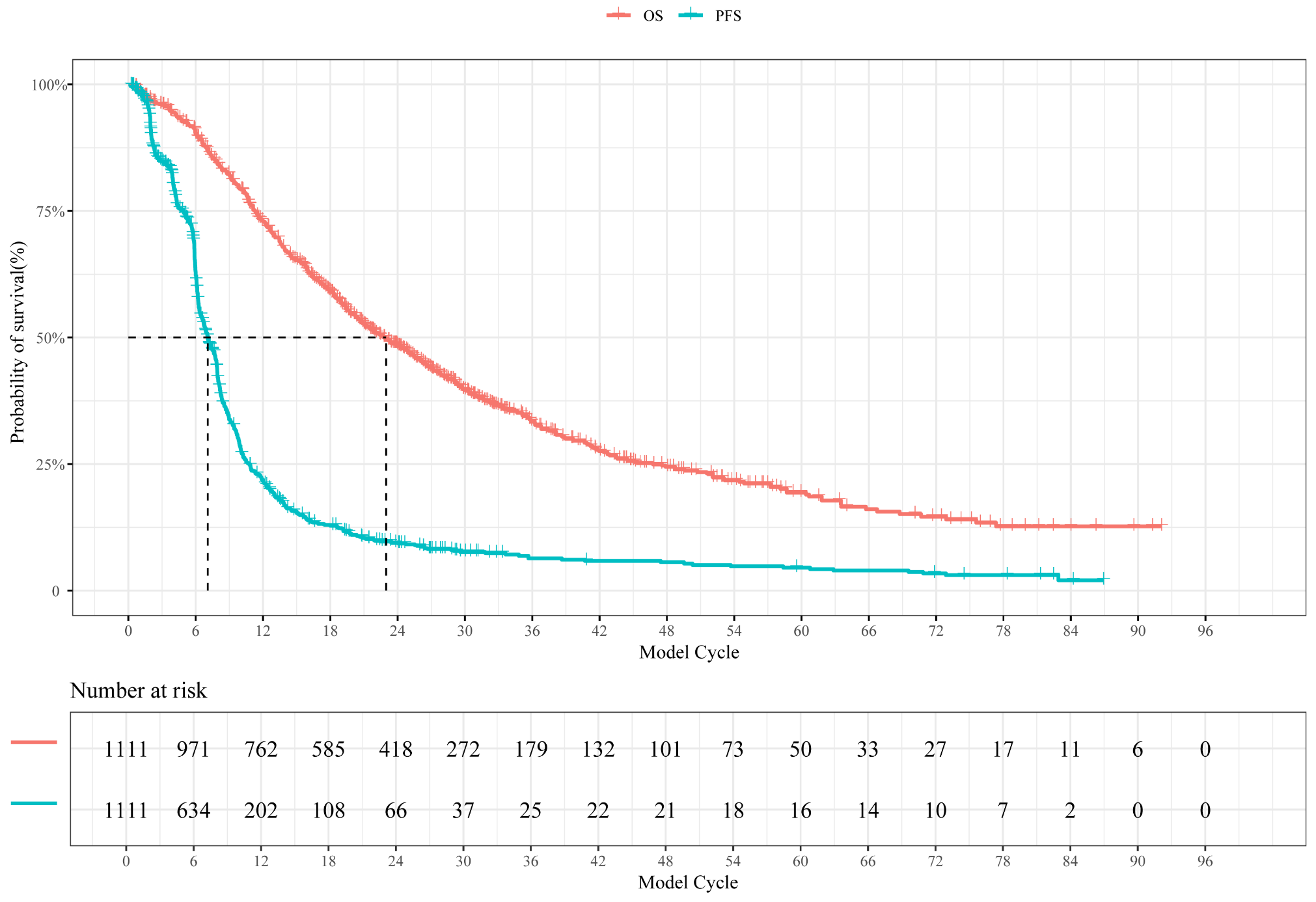


Supplementary Figure 4. The reconstructed Kaplan-Meier OS and PFS curves based on the pooled time-to-event data of chemotherapy regimen.

Abbreviations: PFS progression-free survival, OS overall survival.


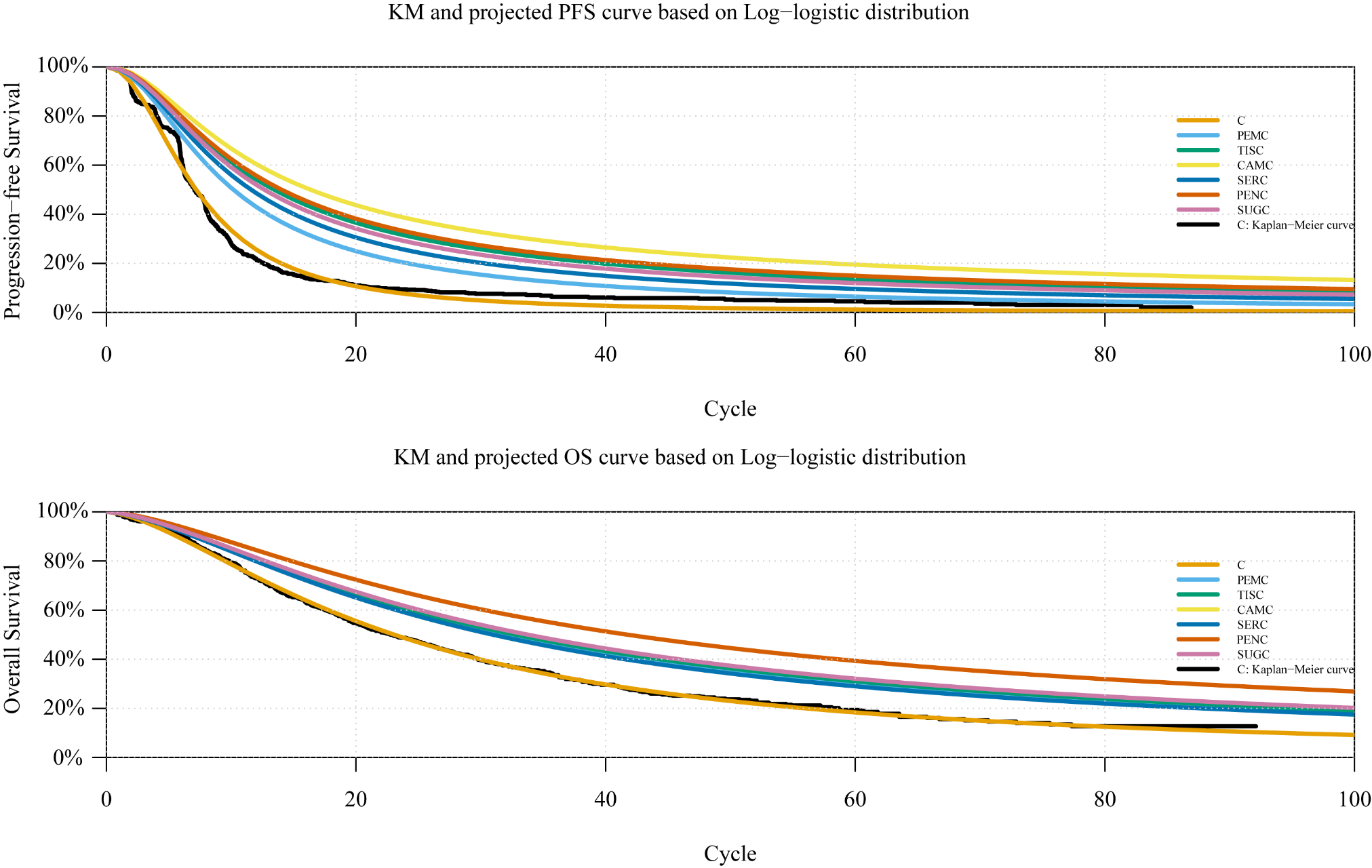


Supplementary Figure 5. Diagram of projected PFS and OS fit curves based on the hazard ratio of network meta-analysis.

***Notes:*** The colored lines represent the projected curves of treatment regimens. The black lines represent the reconstructed Kaplan-Meier survival curves of chemotherapy regimen. Each cycle of the x-axis is three weeks.

***Abbreviations: KM*** Kaplan-Meier, ***PFS*** progression-free survival, ***OS*** overall survival, ***C*** chemotherapy, ***PEMC*** pembrolizumab plus chemotherapy, ***TISC*** tislelizumab plus chemotherapy, ***CAMC*** camrelizumab plus chemotherapy，***SERC*** serplulimab plus chemotherapy, ***PENC*** penpulimab plus chemotherapy, ***SUGC*** sugemalimab plus chemotherapy.


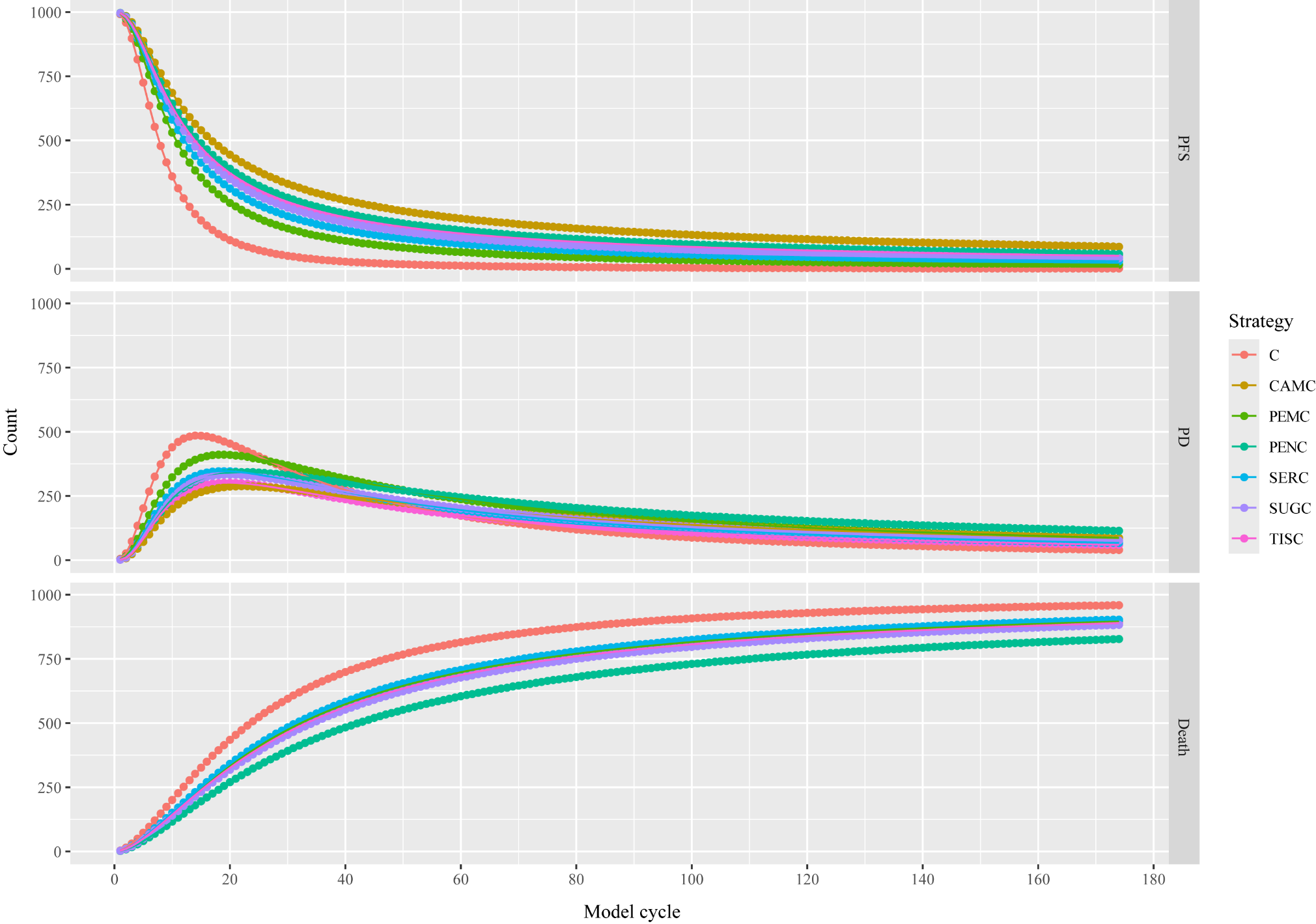


Supplemental Figure 6. Proportion of patients at each time point.

***Notes:*** The colored lines represent the treatment regimen. Each cycle of the x-axis is three weeks.

***Abbreviations:*** ***PFS***, progression-free survival; ***PD***, progressed disease; ***C*** chemotherapy, ***PEMC*** pembrolizumab plus chemotherapy, ***TISC*** tislelizumab plus chemotherapy, ***CAMC*** camrelizumab plus chemotherapy，***SERC*** serplulimab plus chemotherapy, ***PENC*** penpulimab plus chemotherapy, ***SUGC*** sugemalimab plus chemotherapy.

Reference:

Fessas, P., Lee, H., Ikemizu, S., and Janowitz, T. (2017). A molecular and preclinical comparison of the PD-1–targeted T-cell checkpoint inhibitors nivolumab and pembrolizumab. *Seminars in Oncology* 44, 136–140. doi: 10.1053/j.seminoncol.2017.06.002

Gordeev, A., Vaal, A., Puchkova, M., Smirnova, I., Doronin, A., Znobishcheva, A., et al. (2024). Preclinical comparison of prolgolimab, pembrolizumab and nivolumab. *Sci Rep* 14, 23136. doi: 10.1038/s41598-024-72118-3

Ho, C.-L., Chao, T.-Y., Chang, C.-L., and Lin, H.-Y. (2024). Safety, Tolerability, and Preliminary Efficacy of Serplulimab, a Novel Anti-PD-1 Antibody, in Patients with Metastatic or Recurrent Solid Tumors: A Phase I Study. *BioDrugs* 38, 287–299. doi: 10.1007/s40259-023-00639-w

Huang, Z., Pang, X., Zhong, T., Qu, T., Chen, N., Ma, S., et al. (2022). Penpulimab, an Fc-Engineered IgG1 Anti-PD-1 Antibody, With Improved Efficacy and Low Incidence of Immune-Related Adverse Events. *Front. Immunol.* 13, 924542. doi: 10.3389/fimmu.2022.924542

Hutchins, B., Starling, G. C., McCoy, M. A., Herzyk, D., Poulet, F. M., Dulos, J., et al. (2020). Biophysical and Immunological Characterization and *In Vivo* Pharmacokinetics and Toxicology in Nonhuman Primates of the Anti-PD-1 Antibody Pembrolizumab. *Molecular Cancer Therapeutics* 19, 1298–1307. doi: 10.1158/1535-7163.MCT-19-0774

Jiang, M., Liu, M., Liu, G., Ma, J., Zhang, L., and Wang, S. (2023). Advances in the structural characterization of complexes of therapeutic antibodies with PD-1 or PD-L1. *mAbs* 15, 2236740. doi: 10.1080/19420862.2023.2236740

Wang, M., Wang, J., Wang, R., Jiao, S., Wang, S., Zhang, J., et al. (2019). Identification of a monoclonal antibody that targets PD-1 in a manner requiring PD-1 Asn58 glycosylation. *Commun Biol* 2, 392. doi: 10.1038/s42003-019-0642-9

Xu, B., and Sun, H.-C. (2022). Camrelizumab: an investigational agent for hepatocellular carcinoma. *Expert Opinion on Investigational Drugs* 31, 337–346. doi: 10.1080/13543784.2022.2022121

Yang, X., Wang, F., Zhang, Y., Wang, L., Antonenko, S., Zhang, S., et al. (2015). Comprehensive Analysis of the Therapeutic IgG4 Antibody Pembrolizumab: Hinge Modification Blocks Half Molecule Exchange In Vitro and In Vivo. *Journal of Pharmaceutical Sciences* 104, 4002–4014. doi: 10.1002/jps.24620

Zhang, L., Geng, Z., Hao, B., and Geng, Q. (2022). Tislelizumab: A Modified Anti-tumor Programmed Death Receptor 1 Antibody. *Cancer Control* 29, 10732748221111296. doi: 10.1177/10732748221111296

Zhou, C., Wang, Z., Sun, Y., Cao, L., Ma, Z., Wu, R., et al. (2022). Sugemalimab versus placebo, in combination with platinum-based chemotherapy, as first-line treatment of metastatic non-small-cell lung cancer (GEMSTONE-302): interim and final analyses of a double-blind, randomised, phase 3 clinical trial. *The Lancet Oncology* 23, 220–233. doi: 10.1016/S1470-2045(21)00650-1
